# The low spike density of HIV may have evolved because of the effects of T helper cell depletion on affinity maturation

**DOI:** 10.1371/journal.pcbi.1006408

**Published:** 2018-08-30

**Authors:** Assaf Amitai, Arup K. Chakraborty, Mehran Kardar

**Affiliations:** 1 Department of Chemical Engineering, Massachusetts Institute of Technology, Cambridge, Massachusetts, United States of America; 2 Institute for Medical Engineering and Science, Massachusetts Institute of Technology, Cambridge, Massachusetts, United States of America; 3 Ragon Institute of MGH, MIT and Harvard, Cambridge, Massachusetts, United States of America; 4 Department of Physics, Massachusetts Institute of Technology, Cambridge, Massachusetts, United States of America; University of Texas at Austin, UNITED STATES

## Abstract

The spikes on virus surfaces bind receptors on host cells to propagate infection. High spike densities (SDs) can promote infection, but spikes are also targets of antibody-mediated immune responses. Thus, diverse evolutionary pressures can influence virus SDs. HIV’s SD is about two orders of magnitude lower than that of other viruses, a surprising feature of unknown origin. By modeling antibody evolution through affinity maturation, we find that an intermediate SD maximizes the affinity of generated antibodies. We argue that this leads most viruses to evolve high SDs. T helper cells, which are depleted during early HIV infection, play a key role in antibody evolution. We find that T helper cell depletion results in high affinity antibodies when SD is high, but not if SD is low. This special feature of HIV infection may have led to the evolution of a low SD to avoid potent immune responses early in infection.

## Author summary

The spike protein on the virus surface mediates its entry to the host cell and a high spike density promotes infection. HIV has a spike density that is almost two orders of magnitude lower than other viruses. This unique feature of HIV has defied explanation since it was first observed. By bringing together theory and computation, rooted in statistical mechanics, with immunology we suggest that the effects of dramatic depletion of T helper cells during HIV infection on antibody production provided an evolutionary driving force for HIV to evolve a low spike density in order to avoid potent immune responses. Additionally, we show that an intermediate spike density induces maximally potent antibody production, a result with implications for vaccine design.

## Introduction

Viruses gain entry into their host cells by attaching to specific receptors on the host surface. The proteins that mediate entry comprise the viral spike. Since the host receptor does not mutate rapidly, spike proteins, while often being highly mutable, have conserved regions that bind to elements on the host receptor. For example, the HIV spike protein gp120 contains relatively conserved residues that bind to the CD4 co-receptor on T helper cells. In influenza, the spike is composed of a HA glycoprotein, that attaches to sialyl-oligosaccharide, which is a sugar found in many cell surface proteins [1].

From the standpoint of mediating cell entry and thus propagating infection, it is evolutionarily beneficial to exhibit a high concentration of spikes on the virus surface, thus increasing the probability of attaching to host cell receptors [2]. But, parts of the proteins that comprise the viral spikes are also the targets (epitopes) of antibodies produced by the humoral immune response. A lower spike density (SD) would hinder antibodies from binding to the same epitope on two spikes on the viral surface simultaneously with its two arms, thus taking advantage of cooperativity of binding by the two arms (avidity) [3]. Thus, there is also an evolutionary driving force for viruses to evolve a low SD. But, evasion of potent immune responses may not always favor a low SD. For example, in influenza, hypervariable features at the head of the spike have high immunogenicity. A high SD protects the more conserved domains near the stem from being targeted by antibodies [4]. In HIV, the conserved regions are partially protected from the action of antibodies by a shield of glycans or by their membrane-proximal location (a high SD would presumably better shield the latter epitopes). Furthermore, many immunogenic epitopes that do not include any conserved residues are also present on the HIV spike.

Available data indicate that most viruses express a very high number of spikes on their surface. For example, Influenza has around 450 spikes or a SD of 1 spike per 100 nm^2^ [5], HCV has 250 spikes or SD of 1.73 per 100 nm^2^ [6]. Table 1 summarizes much of the available information on SDs of common viruses, and this data shows that HIV is an extreme outlier, exhibiting between 7 and 14 spikes on its surface, resulting in a very low SD of 0.01 spikes per 100 nm^2^, which is 50–100 times lower than that for other viruses [7]. So, it appears that most viruses evolved high SDs (presumably enhancing infectivity and possibly distracting the immune system from targeting more conserved epitopes), while HIV has not. If HIV has evolved a low SD to avoid potent antibody responses, why have other viruses not employed the same strategy? If HIV spike proteins were significantly more vulnerable to antibody responses compared to other viruses, this could explain why HIV has evolved a significantly low SD to lower the avidity of antibody binding to epitopes on the spike proteins. But, no evidence exists suggesting that this is true. Another possibility would be that HIV as a retrovirus, has some fundamental architecture constraints on the maximum number of spikes it can display. However, MLV (another retrovirus) has a high surface density [8] with at least a hundred spikes on its surface [9]. Shedding of the spike (gp120) as an immune decoy could be another reason for the low spike number. However, high spike density viruses such as Ebola also shed their glycoprotein spikes [10]. Hence, this mechanism is not sufficient to explain HIV’s uniqueness of low spike density. Thus, an obvious long-standing question remains unanswered: why has HIV evolved to exhibit a significantly lower SD compared to other viruses?

**Table 1:** Diameter and spike density for different viruses. See S1 Text—“Spike density of different viruses” for details.

Virus	Diameter [nm]	Spike number per virion	Surface density [spikes per 100 nm2]	Reference
Influenza A	120	450	1	[5]
Herpes Simplex Virus	186	659	0.6	[62]
Dengue	41	60	1.13	[63]
Zika	60	60	0.53	[64]
Hepatitis C virus (HCV)	73	290	1.73	[6]
Human immunodeficiency virus (HIV)	120	7–14	0.01	[7,43]

Upon infection with pathogens, high affinity antibodies develop by a Darwinian evolutionary process called affinity maturation (AM). We inquired if the biology of HIV may influence the effects of SD on AM in a way that is not characteristic of other viruses, and whether this is the underlying reason for a low SD being favored by HIV. To explore this possibility, we developed a coarse-grained computational model of the dynamics of AM.

Results of our calculations show that antibody affinity to the epitopes on the viral spike is a function of its SD for all cases. In particular, highest affinity antibodies are produced for an intermediate SD. To the best of our knowledge, this effect of SD on the resulting Ab affinity has not been reported before. Importantly, we find that the decline in antibody affinity when SD exceeds the optimal value is very gradual, while the affinity declines sharply for SDs below the optimum density. These results suggest that a high SD (beyond the optimum defined above) allows viruses to exhibit high infectivity and evade potent responses directed toward mutationally vulnerable epitopes if they are located at the stem of the spike, while also reducing the affinity of antibodies directed toward the spike. Note, however, that this still allows the immune system to generate reasonably high affinity antibodies as the decline in affinity for SDs higher than the optimum is gradual.

Why is HIV different? Our calculations suggest that the answer lies in a key feature of HIV infection. HIV principally infects T helper cells (CD4 T cells). We find that if T helper cells become extraordinarily limiting during affinity maturation, as is the case immediately following HIV infection, then high spike densities will elicit even higher affinity antibodies—a bad outcome for the virus. We find that this is circumvented if the SD is low. Therefore, our results suggest that a key benefit of a lower SD for HIV is an avoidance of high affinity antibody responses that would otherwise be produced if the SD was high when T helper cell availability becomes much more limiting than usual. However, the tradeoff for having low SD is reduced infectivity [11], a long noted feature of this virus. The virus’s low infectivity has not prevented HIV transmission from reaching epidemic proportions; perhaps, because of its high replication rate in infected hosts.

## Results

### Model description

**Fig 1:**
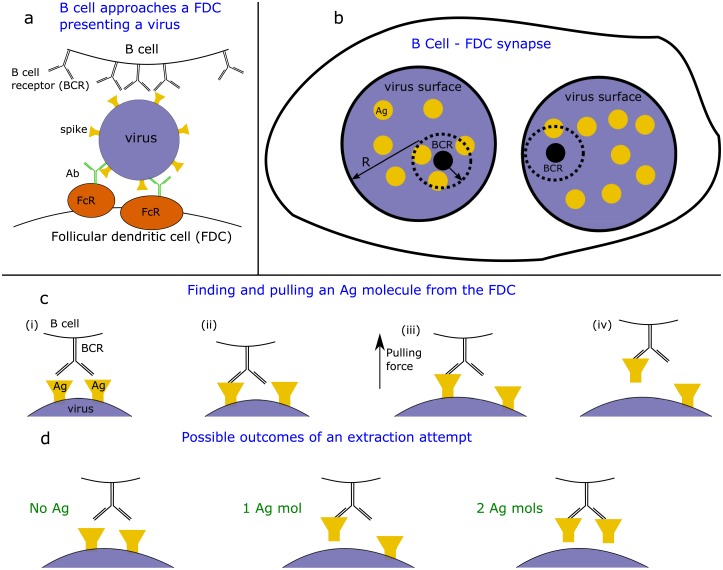
Extraction of Ag molecules from an immune complex on an antigen-presenting cell. **(a)** A B cell approaches a FDC in order to capture antigen (Ag). The FDC presents an immune complex holding a virus (purple) whose surface is covered by spike proteins (yellow), which are the Ags. The attachment of the virus to the FDC is mediated by low-affinity antibodies (green) bound to receptors, called Fc Receptors (orange, FcR), on the FDC surface. **(b)** Schematic representation of the synapse between the B cell and the FDC (top view). The apparent virus surface is of radius R, where Ag (spike) molecules are distributed. The BCR (black) scans a region, attempting to capture Ag. (**c**) The extraction of antigen molecules from a FDC by a B cell: **(c-i)** A protrusion containing the BCR extends to touch the surface of the FDC displaying Ag molecules. **(c-ii)** Depending on the Ag concentration on the FDC, the BCR may encounter Ag molecules to which it can attach. **(c-iii)** Once a BCR-Ag bond has formed, the B cell pulls on it by applying force mediated by actin. **(c-iv)** Extraction of Ag from FDC can occur. **(d)** Three possible outcomes to the extraction attempt: The initial bond between the BCR and the Ag is severed and no Ag is extracted; the BCR bond holds while the IC-Ag bond breaks and one Ag molecule is extracted; the second arm finds another Ag before the first IC-Ag bond breaks and two Ag molecules are extracted. A scenario where the entire virus+Ab is extracted from the FDC is also feasible and gives similar results in our model.

Antibodies develop in domains within secondary lymphoid organs called germinal centers (GCs), which appear shortly after infection [12]. B cells with a moderate threshold affinity for the antigen (Ag) are activated and seed GCs. These B cells then undergo an evolutionary process of mutation and selection that results in B cells with higher affinity receptors as time ensues [13]. The AID gene introduces mutations into the B cell receptor (BCR) at a high rate in GCs. The mutated B cells undergo selection against Ag, which is displayed on Follicular dendritic cells (FDC) on immune complexes (IC) [14] (Fig 1a). The B cells attempt to capture Ag by forming transient synapses with the FDCs [15]. Captured Ag is processed and presented on their surface as peptide-MHC class II complexes. The B cells then compete with each other to bind to T follicular help cells (TfhCs) via interactions between these peptide-MHC complexes and the T cell receptor on the surface of TfhCs. Successful binding results in a survival/proliferation signal. B cells that display more peptide-MHC complexes have an advantage in this competition[16]. The majority of positively selected B cells undergo further rounds of mutation and selection [17–19]. Some of the positively selected B cells differentiate into antibody-secreting plasma cells and memory cells. As time progresses, antibodies with increasing affinity for the Ag are generated.

Affinity maturation has been studied extensively using theoretical models over the last decades, mostly using population dynamics approaches [19–24] and more recently, using detailed simulation of the dynamics of the immune cells invovled in the GCR [25]. To explore how B cell selection and the generation of high affinity Abs depend on Ag concentration and presentation, we constructed a simplified model of Ag capture from the FDCs, and a coarse-grained model of B cell selection in a GC. Thus, our model generalizes previous modeling approaches to include the interaction of the BCR with Ag (spike).

### Antigen distribution on FDCs

We are interested in ICs presenting a virus, or a virus fragment (Fig 1a). The Ags of interest are the spike proteins distributed on the virus surface. Assuming that the spikes are distributed on the surface of the virus of radius *R*, with average density *n*_*Ag*_, it contains a total of *M*_*Ag*_ = 4*πR*^2^*n*_*Ag*_ spikes (Fig 1b). A virion particle with a radius of 120nm (Table 1) with spike density of 0.35 spikes/100nm^2^, has about *M*_*Ag*_ = 160 spikes on its surface. During the Ag capture process, BCRs scan for Ag over a region of the synapse and encounter an Ag molecule with probability *p*. We assume that the number *N*_*Ag*_ of Ag molecules in the scanning area is distributed randomly, according to the Binomial distribution (*N*_*Ag*_ ~ *B*(*M*_*Ag*_, *p*)).

### Ag encounter and internalization by the BCR

Consistent with data showing that B cell protrusions that extended toward FDCs retract if the associated BCRs do not find Ag [26], we assume that the BCR has a characteristic time to find the Ag (see Methods). If it does not bind to Ag during that time, no Ag is captured (see Fig 1ci and 1cii). Once one of the arms of the BCR binds to Ag, an Ag molecule may be pulled away by force[27]. At this stage, there is a tug-of-war over the Ag between the BCR and the IC (see Fig 1ciii). Characteristically, when the binding energy between the BCR and the Ag is much larger than the binding energy between the Ag and the virus/Fc receptor/FDC membrane (see Fig 1), the Ag will be captured by the BCR. We denote the potential interaction energy required to extract the Ag by *E*_*Ag*−*mem*_, and the interaction energy of the BCR/Ab and Ag by *E*_*Ag*−*Ab*_. A successful Ag capture event occurs when the bond between the Ag and the membrane ruptures before the bond between the BCR and the Ag (see Methods).

When the off-rate of the BCR arm from the Ag is smaller or of the order of the effective on-rate (*qN*_*Ag*_), and if one arm is attached initially (see Fig 1cii), the second BCR arm can bind to another Ag molecule if one is available. Thus, a pulling attempt can have three possible outcomes, with zero, one or two Ag molecules captured (see Fig 1d), depending on the binding affinity and the number of available and accessible Ag molecules *N*_*Ag*_.

### Simplified model of GC reaction

In the first days following infection or immunization, *M* different clones (unique BCRs) of activated B cells proliferate with little competition, creating a pool of cells on which AM operates[28]. Few or no mutations are introduced to the BCR sequence at this early stage. We use a simple birth/death process to describe this initial growth stage.

B cells then start to mutate, and whether or not a B cell is subsequently positively selected depends on its ability to internalize Ag and compete with other B cells for survival signals by interacting with TfhCs. TfhCs have an important role in regulating the duration of the cell cycle in B cells during AM [17,18]. Following a TfhC signal, B cells divide (and mutate) multiple (4–6) times before going back for another round of selection [29–31].

Most theoretical models enforce selection by eliminating cells with low affinity BCR [23,24]. It has been shown [17] that TfhCs control the rate at which B cells go through the cell cycle. B cells that receive strong proliferation signal from the TfhCs divide multiple times in the dark zone before going back to the light zone. We model this behavior by using a birth-rate for B cells which is proportional to the amount of captured Ag.

The proliferation rate of B cells is a function of the number of captured Ag molecules, further modulated through competition for TfhCs. Indeed, it was shown [32], that TfhCs preferentially interact with B cells that have captured more Ag, presumably giving them a stronger proliferation signal. To mimic this competition we assume the birthrate of B cell *i* to be:

$$\beta_{i}=\beta_{0}\frac{C+A_{i}}{C+\langle A\rangle },$$ (1)

where 〈*A*〉 is the average amount of Ag consumed by all B cells that captured *at least* 1 Ag (B cells that do not capture Ag have zero birth rate); *β*_0_ is the basal birthrate, and *C* is a measure of the availability of TfhC help. When *C*>>〈*A*〉, all B cells get the same amount of metabolic boost and divide at the same rate *β*_0_. When *C*<<〈*A*〉 TfhC help is limiting and the cell birthrate is proportional to the amount of captured Ag. The GC has a limited capacity and grows during this competitive phase according to a stochastic logistic growth process. Thus, the selection process depends on the number of B cells in the system through the logistic death rate (Methods Eq (6)). This logistic term accounts for the increased competition between B cells to receive a survival signal from TfhCs when the number of the latter is limiting. TfhCs are essential for GC maturation of B cells, and without them, AM of B cell cannot occur [33]. We assume here that the number of TfhCs is not so small as to stop the formation of the GC. Thus, at the limit where *C* goes to zero, our model does not corresponds to the absence of TfhCs in the GC. However, we do use the reduction of *C* to model increased B cells competition in a GC when the number of TfhCs is gradually reduced, as occurs during the first stages of HIV infection. For these reasons, we do not consider here the limit of *C*→0. In a more complete proliferation model *C* should depend on the total number of B cell in the GC and thus change with time. However, the qualitative behavior of our model would not change if the parameter *C* would have such explicit dependency on the GC size as upon HIV infection we expect Tfh levels to be smaller at all times than upon infection with other pathogens.

### Affinity change following BCR mutation

During AM, B cells mutate the BCR encoding gene using the AID enzyme. This results in changing cytosine to tyrosine on one DNA strand [34]. We modeled the effect of mutation as a change in the on-rate *q* or the interaction energy *E*_*Ag*−*Ab*_ upon cell division (see Methods). We convert the interaction energy to the off-rate by $r_{0}=e^{-\beta E_{Ag-Ab}}$, and the affinity of the BCR is calculated as *ω* = *q*/*r*_0_. Our simplified model assumes that affinity improvements and reductions (through *q* or *E*_*Ag*−*Ab*_) are equally likely following mutation and that all mutations change affinity. These choices are certainly not realistic, but making advantageous mutations rarer than deleterious ones, or making some of the mutations silent or lethal (as in reality) do not change the qualitative results.

### Highest affinity antibodies are generated at an optimal spike density

**Fig 2:**
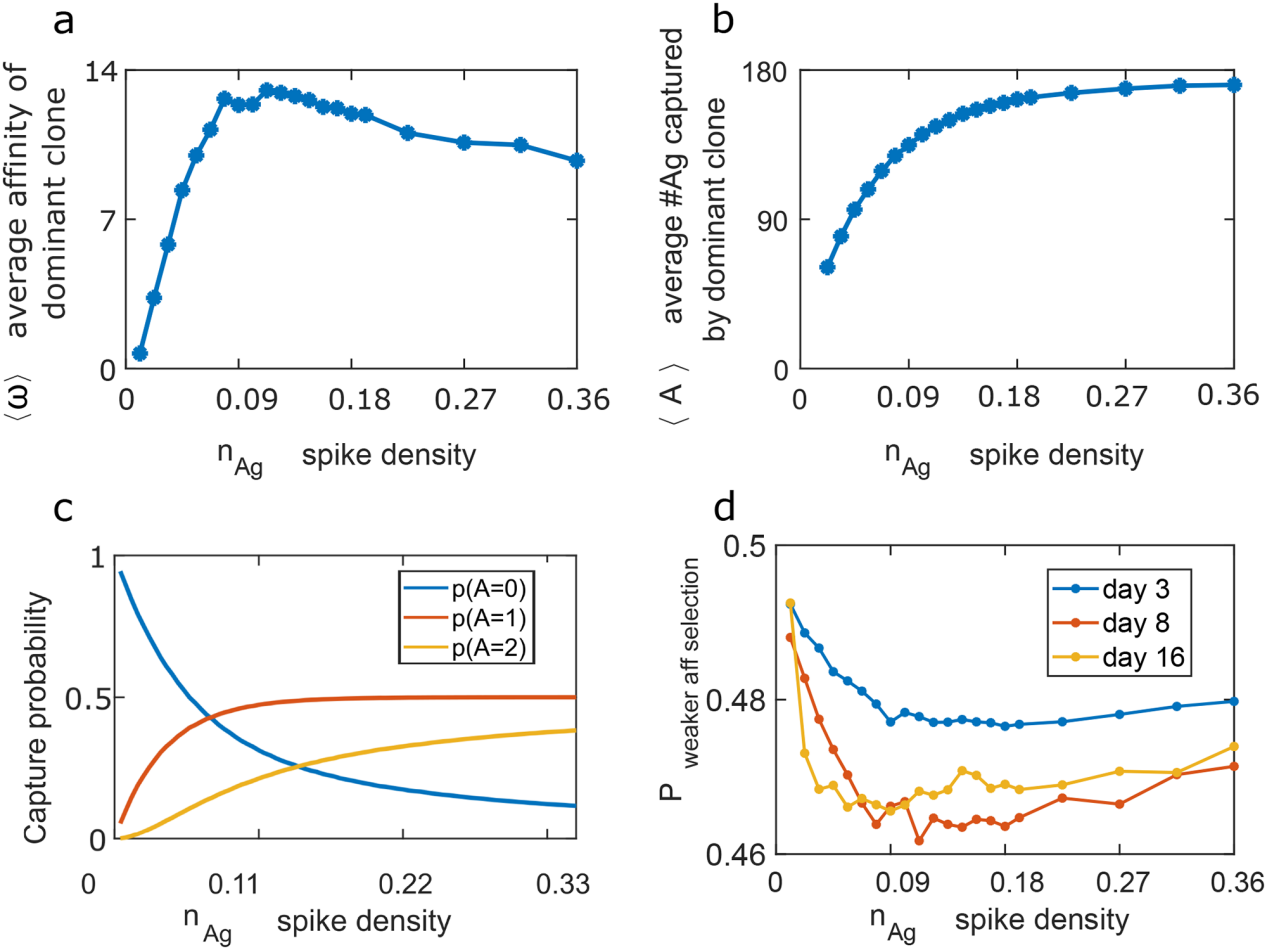
Affinity maturation (AM) in a germinal center (GC). Simulations of the GC reaction starting with 20 different B cell clones. During AM, cells mutate their BCR upon division, changing only the BCR interaction energy with the Ag (*E*_*Ag*−*Ab*_) and thus the off-rate *r*_0_ of the BCR from the Ag. (**a**) The mean affinity (*ω* = *q*/*r*_0_) of the dominant clone at day 16 of the GC reaction in the competitive phase, as a function of spike density *n*_*Ag*_. (**b**) Mean number of Ag molecules captured by the dominant clone at day 16 as a function of the spike density. (**c**) The capture probability of Ag molecules by a single BCR (Eq (S10)) when the binding energy of the BCR is *E*_*Ag*−*Ab*_ = 1.5. The other parameters of the captures process are listed in Table 2. (**d**) The probability of selecting the weaker affinity B cell (see text) during the competitive phase. This probability depends on the spike density (Eq (S11)). We sampled energies of B cell pairs from the population energy distribution (S5 Fig) obtained in the simulation results shown in (a), to estimate the probability of the lower affinity cell proliferating before a higher affinity one, at different times during the GCR. The relative mild variation in this figure are amplified in multiple rounds of selection.

To explore the role of SD on AM, we calculated the affinity of the most dominant clone at the end of the GC reaction (GCR), which is day 16 of the competitive phase. The affinity of this clone will play an important role in the resulting humoral immune response. An important observation is that the affinity of the dominant clone varies non-monotonically with the SD (*n*_*Ag*_). Fig 2a demonstrates this finding for a TfhC level that was limiting across the range of *n*_*Ag*_ (SD) spanning 0.01 to 0.36. There is also a pronounced asymmetry in the variation of affinity with *n*_*Ag*_. As *n*_*Ag*_ increases from very small values, there is a sharp rise in affinity, while its drop for larger *n*_*Ag*_ is gradual. The number of B cells in the GC is not reduced when SD is high. Since B cells have to capture at least 1 Ag molecule in order to attempt proliferation, the average proliferation rate increases with SD.

These qualitative results are robust to variations in the other parameters in the model (S1 Fig), and for mutations resulting in changes in *q* (S2 Fig) or *E*_*Ag*−*Ab*_ (Fig 2a). (The variation of affinity with SD is less pronounced when mutations change *q* for technical reasons described in S2 Fig.)

In the GC, memory and plasma B cells are produced throughout the GCR [12]. As a proxy for the affinities of memory and plasma B cells produced during the maturation process, we randomly selected 10% of the B cells at each intermediate time point. The resulting average, displayed in (S3 Fig) still exhibits a non-monotonous dependence on SD similar to that observed in Fig 2a.

The mechanistic reason underlying these results relates to the amount of antigen that is internalized by B cells as *n*_*Ag*_ changes (Fig 2b). Upon increasing *n*_*Ag*_ from a small value, the amount of Ag internalized by B cells grows significantly as it becomes more probable to bind one or two Ag molecules (see Fig 2c). However, once the SD is sufficiently high, most BCRs bind/internalize on average the same amount of Ag irrespectively of their affinity (if it is above a threshold value), and it is difficult to distinguish B cells based on the affinity of their receptor for the antigen. Competition between B cells to be positively selected is not severe in the latter regime, limiting the driving force to increase affinity by further mutations. Thus, the affinity of the antibodies produced begins to decline beyond an optimal SD. The gradual drop-off in affinity as *n*_*Ag*_ becomes too high, compared to the sharp decline when *n*_*Ag*_ becomes too low, is because the number of internalized antigens rises very sharply as *n*_*Ag*_ increases from a low value, but then slowly saturates to its maximum for sufficiently high SDs as depicted in Fig 2b. In the limit of high *n*_*Ag*_, there is a finite probability for the BCR to capture 1 or 2 Ag mols. At this limit, the probability to extract one Ag mol is given by *r*/(*r* + *λ*) and the probability to extract two Ag mols is *λ*/(*r* + *λ*), where *λ* is the off-rate of an Ag mol from the membrane when one arm of the BCR is bound and *r* is the rupture rate of the BCR from an Ag mol when one arm of the BCR is bound.

Our model suggests that clonal diversity also varies with SD. When SD is low, antigen is scarce and the selection forces will be fiercer. That could result in a more rapid loss of diversity. We estimate diversity by computing the fraction of the GC comprised of the dominant clone (S2b Fig, S4a Fig), which we denote by *f*_*d*_. For very low SD a few dominant clones comprise most of the GC’s B cells at the end of the GCR (large *f*_*d*_). This is because for low SD, selection during AM is dominated by the large competitive advantage, compared to most B cells, of a few B cells that internalize more antigen due to stochastic fluctuations. As the SD increases, a greater number of B cells can internalize antigen successfully and the GCR produces a more diverse clonal population.

The fraction of dominant clones has a large variability across different GC realizations. Indeed, it has been shown [31] that while some GCs appear to have been taken over by a single clone at day 16, others show high clonal diversity. This is the direct result of stochastic selection forces at play in the GC [35]. A similar behavior is observed in our GC model (see S4 Fig). Here, the source of stochasticity is the random Ag capture process, as well as the stochastic proliferation and death of B cells.

While the fraction of the dominant clone mostly decreases with increasing SD, the higher affinity of the BCR at intermediate density contributes to a small increase in *f*_*d*_. Thus, *f*_*d*_ is not monotonically decreasing with SD. Indeed, the improved selection at an intermediate density leads to greater loss of diversity in this range, which appears as a small bump in *f*_*d*_ (approximately between *n*_*Ag*_ = [0.1, 0.18], see inset S4 Fig). This effect is more pronounced when the overall diversity loss is slower as a result of a smaller death rate (see S1 Fig).

### Selection forces act with the highest fidelity at an intermediate spike density

To understand how cell selection in AM is related to Ag density, we estimated the probability *P*_weaker aff selection_ that, in any given round of mutation and selection, a B cell with a lower affinity for the antigen gets selected and expands in favor of another B cell that has a higher affinity. A B cell with a higher affinity receptor is likely to internalize more antigen, be more successful at obtaining a survival signal from T helper cells and proliferate more. However, because Ag capture is probabilistic, and the birthrate relates to the amount of captured Ag, there is a chance that a B cell with lower affinity will be selected in favor of one that expresses a higher affinity receptor.

To empirically estimate *p*_weaker aff selection_, we sampled B cell pairs from the affinity distribution computed in our simulations (see S5 Fig). We detail how the probability is estimated in the methods section. Interestingly, we find that the probability changes with time, and depends on Ag density (Fig 2d). Importantly, *p*_weaker aff selection_ is minimal at intermediate Ag density, at a value similar to the maximum for the BCR affinities. Thus, very high or low SDs lead to poor differentiation between higher and lower affinity B cells. Because the B cells with a higher affinity are most likely to be chosen to proliferate at an intermediate SD, selection is strongest for such SD, and thus highest affinity antibodies evolve.

### Cooperative binding with two arms of the BCR leads to optimal affinity increase at intermediate densities

To further elucidate the origin of the maximum in antibody affinity at an intermediate SD, we studied the time evolution of the mean affinity of the B cell population. We aimed to find the dependency of the optimal density on the rates of different processes in our model, rather than attempt to recapitulate our simulation results. Assuming that the time *τ* to find the first Ag molecule before retracting the B cell protrusion is very long and that each B cell has only a single BCR, we find a mean-field equation (see SI) for the evolution of the mean on-rate $\bar{q}$

$$\tau_{0}\frac{d\bar{q}}{dt}=\frac{\text{cov}(q,A)}{\langle A\rangle +C_{1}},$$ (2)

where 〈*A*〉 is the average amount of captured Ag by the B cell population (here 〈*A*〉 is between 0 and 2), cov(*q*, *A*) is the covariance between the number of captured Ag molecules *A* and *q*, *C*_1_ is the parameter that corresponds to TfhC availability parameter and *τ*_0_ ∝ (*β*_0_−*μ*_0_)^-1^ is the time scale of the process. We recall Fisher’s fundamental theorem of natural selection stating that “The rate of increase in fitness of any organism at any time is equal to its genetic variance in fitness at that time” [36]. Eq (2) is a generalization of Fisher’s fundamental theorem and is also reminiscent of Price’s equation [37] if we consider *q* to be a *trait* of the population. We further find that the variance *σ*^2^ of the on-rate distribution evolves as

$$\tau_{0}\frac{d\sigma^{2}}{dt}=\frac{\text{cov}\left({\left(q-\bar{q}\right)}^{2},A\right)}{\langle A\rangle +C_{1}}+2D.$$ (3)

Solving Eqs (2) and (3) numerically (S6 Fig), we find that $\bar{q}$ exhibits the same non-monotonic behavior as in our simulation results S2a Fig. When TfhC level is scarce (small *C*_1_), the affinity increases faster due to the increased competition between B cells (S6 Fig). The position of the optimal density changes with time (S6 Fig) towards smaller Ag densities. For a given on-rate distribution (fixed mean and variance), the rate of increase in affinity is maximal at density n* given by

$$n^{*}=\frac{\left(r+\lambda \right)\left(k+\xi \right)}{\bar{q}\xi }\sqrt{\frac{\lambda +C_{1}\left(r+\lambda \right)}{\left(1+C_{1}\right)r+\left(2+C_{1}\right)\lambda }},$$ (4)

where *λ* and *ξ* are the off-rates of the Ag from the IC when one or two arms of the BCR are bound, respectively. *r* and *k* are the BCR arm rupture rate from the Ag when one or two arms of the BCR are bound to Ag, respectively (see SI).

When the interaction energy of the Ab and the Ag is equal to that of the Ag with the IC (*E*_*Ag*−*Ab*_ = *E*_*Ag*−*mem*_), the optimal density is

$$n^{*}=4\frac{\lambda_{0}}{\bar{q}}e^{\beta x_{b}F}\sqrt{\frac{1+2C_{1}}{3+2C_{1}}},$$ (5)

where *λ*_0_ is the disassociation rate of the Ag from the IC. Thus, at the optimal selection density, the characteristic effective on-rate ($n^{*}\bar{q}$) is equal to *λ*_0_. At this density, B cells spilt into different populations, each capturing a different amount of Ag.

Interestingly, for very large values of *q*, the number of captured Ag molecules *A* is independent of *q* as the covariance between them goes to zero (see SI). In this limit, it is impossible to differentiate between B cells affinities and the mean affinity stops increasing, reaching an asymptotic value. Similarly, the variance of the distribution reaches an asymptotic value.

Finally, we speculated whether a scenario where the BCR has one arm (see S7 Fig) would still show the non-monotonic dependence on Ag density. We estimated the time evolution of $\bar{q}$ for a hypothetical BCR with one arm (see SI and S7 Fig). Interestingly, when the time *τ* to find the first Ag molecule is of the same order as the effective on-rate (basal on-rate multiplied by the density), the mean affinity has a maximum at intermediate densities (see S7 Fig). However, *τ* is likely much larger than the on-rate, in which case the affinity is monotonically decreasing as a function of the Ag density (see S7 Fig). We conclude that the optimal SD observed in our simulations is a direct result of the *cooperativity* between the two arms of the BCR because the analytical model shows that an optimal SD emerges only when the BCR has two arms. The cooperativity allows a second arm to search and bind an Ag molecule while the first is bound to another Ag molecule on the surface of the IC.

### Ag depletion improves AM at high SD

During AM, Ag is depleted from IC as it is being consumed by the B cells. As shown in the previous sections, AM depends on the ability of the GCR to differentiate bewteen B cells of different affinities. Optimal selection is achieved when only B cells with the highest affinity are likely to capture 2 Ag molecules (see Eq (5)). Depletion of Ag from FDCs during AM will result in fewer immune complexes encountered by the BCRs. It will not change the local SD on the virus. Rather, it will reduce the first encounter probability of Ag molecules by a BCR (See Eq (7)). To study how the competative forces may be modulated in time, we studied a hypothetical scenario where SD exponentially decays during AM ($n_{Ag}(t)=n_{Ag}(0)e^{-t/T_{Ag-decay}}$). Interestingly, when the SD decays, selection improves when the initial SD is high (S8 Fig). As a result, the affinity at day 16 of the GCR is higher compared to the fixed SD scenario (Fig 2a). At high SD, as the affinity of the B cell population improves, decreasing SD allows the GCR to remain at close to the optimal differention point. However, decreasing Ag density harms the development of GCs for which the initial SD is low. Since B cell have to capture at least one Ag mol to proliferate, most GCs do not succeed in reaching day 16 (S8 Fig) in this limit.

### Depleted TfhC numbers during HIV infection affects AM in a way that favors a low virus SD

**Fig 3:**
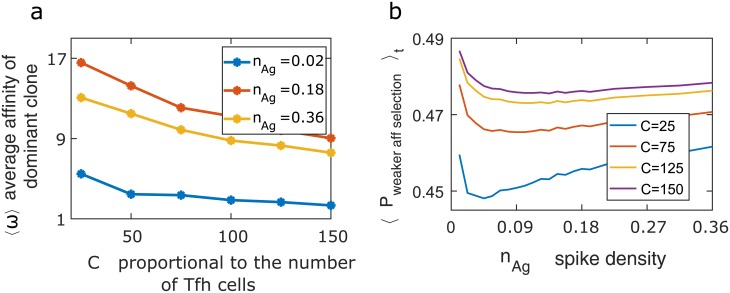
Effect of T helper cell level and spike density on affinity maturation. (**a**) The average affinity of the dominant clone in a GC increases for viruses exhibiting a high spike density as Tfh levels (*C*) decline. Different curves correspond to different values of spike density (*n*_*Ag*_). *E*_*Ag*−*Ab*_ changes upon mutation in these simulations. (**b**) The probability of selecting the wrong B cell (see text) is shown for different values of *C*. The probability shown is a time average over all GCR days.

HIV dominantly infects CD4 T cells and significantly reduces their number immediately following infection during a time when the first antibody responses are developing in the host [38]. We therefore explored the effects of making TfhC activity even more limiting (than what is usual). To do so, we changed the parameter *C* (Eq (1)), which represents the availability of TfhCs during AM. Again, we use the affinity of the dominant clone at the end of the GCR as a metric of the affinity of antibodies generated. Previously (Fig 2a) we found that for high SDs, antibody affinity was lower than optimal. This was because, for sufficiently high SD, most BCRs internalized one or two antigens and so competition between B cells was restricted. But, when the level of TfhC (*C*) is even more limiting, even for high SDs, small differences between B cells with regard to the amount of internalized antigens are amplified by the intense competition between B cells for interacting with, and receiving a survival signal from TfhCs (Fig 3a, S9 Fig). Thus, if the SD is high, more potent antibodies are generated as TfhC levels decline. However, when the SD is low, decreasing *C* does not alter the affinity of the resulting antibodies significantly. Consistent with this result, the probability of selecting the lower affinity B cell decreases as *C* decreases (see Fig 3b). This is because selection is a stronger force when there are fewer TfhCs. In agreement with our result in Fig 3a, this enhancement in selection forces is more pronounced at intermediate and high SDs compared to low SDs. Thus, as TfhC levels are smaller, high affinity antibodies can be generated more readily for high SDs. Thus, perhaps, HIV, which is the only virus that principally targets T helper cells and depletes their numbers, evolved a low SD to prevent strong antibody responses from developing during the early stages of infection when T helper cell levels rapidly decline. (Note that Human T cell Leukemia viruses also infect T cells, but they do not result in a sharp decline in T helper cell numbers [39]. Their effect on the number and functioning of TfhCs is still unclear [40]).

## Discussion

Proteins that comprise the spikes on the surface of viruses bind to receptors on host cells to propagate infection. A high SD increases the probability of encounters with the host cell’s receptor, thus enhancing infectivity [41]. At the same time, the viral spike is the target of immune attack by antibodies [42] and a high SD may make the virus more susceptible to neutralization. For example, HIV’s low SD can inhibit effective neutralization [3]. The average inter-spike distance for HIV is about 23nm, while the average distance between the arms of the Ab is 15nm. Thus, the two Ab arms are unlikely to bind simultaneously to two Ag molecules, which would decrease avidity [3]. In some viruses like influenza, however, hypervariable features near the head of the spike have high immunogenicity, and a high SD can serve to protect the virus from antibodies that could target more conserved epitopes in the stem of the virus [4]. Thus, there appear to be evolutionary forces that favor both a high and low virus SD. Yet, most viruses have very high SD, and HIV appears to be unique in that its SD is about two orders of magnitude lower (see Table 1).

In order to shed light on the evolutionary forces that may have led to the evolution of a low SD for HIV, we studied a simplified computational model of AM. We found that the affinity of Abs produced by GC reactions is maximal for an intermediate SD (Fig 2a). For very low SD, most B cells internalize a single antigen molecule by binding via a single arm of the BCR or internalize no antigen at all. The occasional B cell that internalizes Ag by chance quickly evolves to become the dominant clone during AM. Thus, for very low SD, fluctuations prevent the system from being in the strong selection regime, thus resulting in lower affinity antibodies. For a high SD, BCRs on B cells are very likely to internalize one or two Ags, and so soon after AM ensues, most B cells internalize quite a bit of antigen. Therefore, there is reduced competition between B cells for obtaining a survival signal from TfhCs, and thus a low driving force to evolve higher affinity BCRs. An intermediate SD results in strong selection forces, and the highest affinity antibodies.

The basis for the optimal affinity at intermediate SD being related to the ability to differentiate between B cells with different affinities during the GC reaction is made clear by another aspect of our results. We show that the non-monotonic dependence of antibody affinity with SD is directly related to the binding cooperativity between the two arms of the BCR. Indeed, for a hypothetical single arm BCR, the affinity of the resulting Abs monotonically decreases with SD (S7 Fig). For normal BCRs with two arms, at the optimal density, the second arm serves to split the B cell population into those who manage to capture the additional Ag molecule while the first arm is still bound, and those who do not. Thus, resulting in strong selection for the B cells that internalize two antigens per BCR.

We thus hypothesize that having two arms may be beneficial for optimal B cell selection in the GC. Obviously, the two arms allow Abs to bind Ag with high avidity. However, the two arms also allow for a more precise differentiation between B cells. B cells usually capture Ag using BCR clusters [26] that can function as a “multiple arms” BCR for the purpose of differentiation. However, perhaps it is most beneficial for the BCR to have two arms (in the context of GC selection) when Ag density is very low.

**Fig 4:**
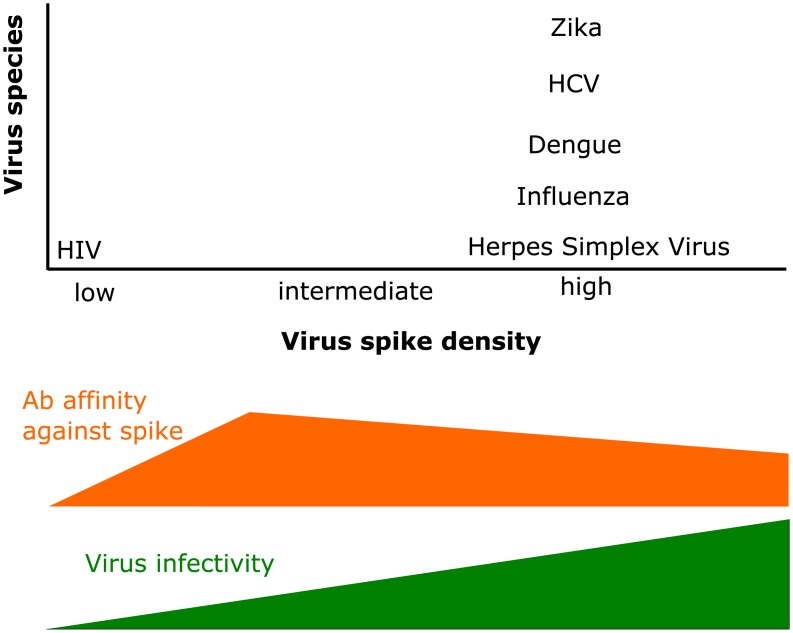
The immune pressure on a virus may determine its spike density. The distribution of viruses species according to their spike density (see Table 1). We illustrate the immune pressure created by Ab affinity (orange), which is non-monotonous with density. The infectivity of a virus increases with its spike density (green).

The probability that a lower affinity B cell is stochastically selected to proliferate in favor of a higher affinity B cell is minimal for the intermediate densities. In other words, the ability of the GC reaction to produce highest affinity Abs at an intermediate SD is because this is the regime where selection effects are strongest. So, viruses could have evolved either a low or high SD to reduce the efficacy of antibodies directed against them. The results in Fig 4 suggest that evolving a low SD would be especially advantageous from this perspective. Furthermore, such a strategy reduces the avidity of antibodies, further inhibiting neutralization [43]. However, most viruses have evolved a high SD (Table 1). Our results suggest that this may be because the evolutionary driving forces of increasing infectivity and shielding conserved epitopes by maintaining a high packing density may be dominant. A very high SD (past the optimal density for AM) can lower antibody affinity somewhat (Fig 4) while favoring these two selection forces.

Why has HIV evolved SD that is roughly two orders of magnitude smaller than that observed for most viruses? HIV is unique in that it principally infects T helper cells and reduces their numbers significantly during the early stages of infection. We suggest that T helper cell depletion during HIV infection results in increased competition between B cells during AM. When T helper cells are limiting to the normal extent, once the SD is higher than the optimal value, selection forces are weaker. But, if T helper cells are significantly depleted, selection forces remain strong at high SDs, resulting in high affinity antibodies. As Fig 3b shows, depletion of TfhCs results in a lower probability of low-affinity B cells stochastically proliferating in favor of higher affinity B cells. However, our results show that, for low SDs, Ab affinity hardly increases upon depletion of TfhCs. This may be the reason that HIV, which is the only known virus that principally infects T helper cells and sharply depletes their numbers during early stages of infection, is the rare virus that has evolved a low SD. The low SD may aid HIV to avoid effective antibody responses in the early stages of infection in a way that would not be possible if it had a high SD. We note also that a low SD can decrease the ability to form diverse clonal lineages during the GC reaction, thus inhibiting AM from deeply exploring the antigenic space upon infection [44]. Because it is a chronic infection and replicates fast, the reduced infectivity of a low SD may be alleviated.

The Measles virus also infects T helper cells; could result in their depletion [45] and causes transient immunosuppression [46]. Unlike HIV, Measles causes acute disease and is rapidly cleared from the body. Notably, Measles has many spikes on its surface [43]. We hypothesize that its rapid mode of propagation resulted in its choice to have high infectivity, producing many virions in the short period during which the patient is infectious. An HIV carrier, on the other hand, is infectious for an extended period. Thus, the virus has to hide for much longer from the immune system and having a low spike density would allow it to do so. Finally, it seems that affinity maturation and antibody response is not the main way by which Measles is cleared. Rather, it induces a T cell response that is dominant in the first two weeks [47]. Only at a much later times (many weeks) affinity maturation starts to produce antibodies against Measles’s RNA and proteins [47]. Another virus that infects T helper cells is HTLV-1. Contrary to HIV, HTLV-1 infection does not appear to result in a sharp decline of the T-cell population but it may impair the function of TfhCs [48]. Our model would suggest that this effect should promote the evolution of a low SD. Another feature of HTLV suggests that a high SD for improving infectivity may be ameliorated; HTLV-1 predominantly infects new cells via cell-to-cell contact [49][50]. It seems that during cell-to-cell transfer, at the intersection between the two cells, the local density of env is high [49]. However, while we could not find precise quantification of their number on the free virions (which is the key variable during affinity maturation), spikes appear sparse on their surface when imaged with cryo-EM [51]. Given this paucity of quantitative data, at this stage, it is not clear how our hypothesis relating spike density and T cell number to the competitive force in the GC should be applied to HTLV-1.

The impact of SD on HIV and SIV propagation has been studied experimentally. A deletion in the tail of gp41 (part of the Env spike protein) has been suggested to increase the number of spikes [41] and their mobility [52]. These mutated virions have better infectivity in cell culture [53]. Surprisingly, the deletion is rarely seen in vivo and when macaques are infected with a short tail SIV mutant, the mutant reverts back to the long tail virus [53–55]. These results may suggest an evolutionary driving force, in the face of an immune response, to reduce SD in HIV.

Our results are reminiscent of the pioneering discovery by Herman Eisen showing that affinity increase upon AM was smaller for a very high Ag dose, suggesting that too high Ag concentration decreases competition in the GC. There is also experimental evidence that intermediate levels of antigen density lead to the highest Ab titers [56], related to optimal BCR activation. In this case, Ag molecules can mediate the formation of a BCR cluster when the density is sufficiently high, while for very high density, Ag sequesters the BCR molecules and the number of fully formed clusters is reduced.

Our results are relevant to vaccine design. It is possible to design liposomal nanoparticles that display varying density of Ag [11,57,58]. We have shown here that more is not necessarily better. We suggest that a precise design of the density of Ag can impact B cell selection in the GC, and as a result on the Ab affinity of the memory and plasma cells that are the product of vaccination. Thus, our results may guide the design of vaccination vectors that could optimize immune responses in the lymph node follicle.

Our arguments regarding the evolutionary driving forces that have led to HIV being unique in exhibiting an extraordinarily low SD may be difficult to examine experimentally (as is the case for most problems in evolutionary biology that are not contrived laboratory curiosities). However, by depleting TfhCs in mice to different degrees, the veracity of the underlying mechanism could be tested.

## Methods

### The growth of the GC

To account for the limited capacity of a GC during the competitive phase, we employed a variant of the stochastic logistic growth process [59], in which the death rate increases with the overall population size, from a basal rate of *μ*_0_, as

$$\mu (\text{n})=\left(\mu_{0}+\left(\beta_{0}-\mu_{0}\right)\frac{\sum_{i=1}^{M}n_{i}}{N}\right).$$ (6)

Here, *N* is the population capacity taken to be 200; **n** = (*n*_1_, *n*_2_,…,*n*_*M*_) is the vector of cell numbers *n*_*i*_ for the *M* clones such that $\sum_{i=1}^{M}n_{i}$ is the total number of B cells in the GC. The competitive phase lasts about 16 days in mice [31]. The total number of cells in the GC grows gradually until reaching the capacity *N*, where it remains approximately fixed.

### Ag encounter and binding by the BCR

We assume here that an arm of a BCR at the tip of a B cell protrusion has a characteristic time *τ* to find an Ag molecule, after which the protrusion retracts empty. The probability that an arm of a BCR at the tip of a B cell protrusion finds Ag before time *τ* is

$$P_{binding}:=\text{Probability}\;\{\text{One}\;\text{arm}\;\text{binding}\;\text{before}\;\text{time}\;\tau \}=\left(1-e^{-2q_{N_{Ag}}\tau }\right),$$ (7)

where $q_{N_{Ag}}=qN_{Ag}$ is the on-rate for the BCR to find an Ag molecule given that there are *N*_*Ag*_ of them in its scanning radius, and the sequence-dependent on-rate with which a particular BCR binds to Ag is *q*. This leads to

$$\begin{array}{l} p_{0}=e^{-2q_{N_{Ag}}\tau },\\ p_{1}=1-e^{-2q_{N_{Ag}}\tau }, \end{array}$$ (8)

where *p*_0_ is the probability that no Ag is encountered, while *p*_1_ is the probability that one of the arms encounters an Ag molecule in this time period.

### Ag internalization

In order to capture the Ag, the BCR attached to a protrusion of the B cell applies a pulling force that works against the interactions of the BCR with Ag, and that between the Ag and the virus/Fc receptor/FDC membrane (see Fig 1). We denote the potential interaction energy required to extract the Ag by *E*_*Ag*−*mem*_, and the interaction energy of the BCR/Ab and Ag by *E*_*Ag*−*Ab*_.

The characteristic rupture time depends on the force applied by the B cell [60]. The force *F* does work *x*_*b*_*F* on the bond, reducing its free energy and increasing the rupture rate to $r_{F}=r_{0}e^{\beta x_{b}F}$, where *r*_0_ is the characteristic rate for bond disassociation when no force is present, *x*_*b*_ is the distance at which the bond ruptures, and *β* = *k*_*B*_*T*. The intrinsic rupture rate with no force is estimated by Kramer’s escape from a potential barrier as

$$r_{0}=Ke^{-\beta E_{Ag-Ab}},$$ (9)

where *K* is a coefficient that depends on the shape of the interaction potential [61]. We take *K* = 1, and note that in writing Eq (9) we have assumed that the activation barrier to form the bond is much smaller than the energy gained upon binding (*E*_*Ag*−*Ab*_). *F* is typically of the order of a few pico-Newtons [27]. We assume that each B cell has 100 BCRs but the qualitative behavior of our results does not depend on the number of BCRs. As each BCR can extract 0/1/2 Ag molecules, a B cell can extract between 0 and 200 Ag molecules.

### Affinity change following BCR mutation

We modeled the effect of mutation as a change in the on-rate *q* or the interaction energy *E*_*Ag*−*Ab*_ upon cell division, with one daughter retaining the parent affinity, while for the other daughter:

$$\begin{array}{l} E_{Ag-Ab,daughter}=E_{Ag-Ab,parent}+\text{N}(0,\sqrt{2D}),\\ q_{daughter}=q_{parent}+\text{N}(0,\sqrt{2D}) \end{array}$$ (10)

Here, N is a normal distribution with zero mean and standard deviation of $\sqrt{2D}$, with *D* akin to an effective variability coefficient determining the magnitude of the change in *q* or *E*_*Ag*−*Ab*_. Within this model, the energy, or *q*, can increase or decrease with equal probability at every division. We added a reflecting boundary condition at zero such that *q* is never negative. Since the off-rate scales as $r_{0}=e^{-\beta E_{Ag-Ab}}$, the affinity of the BCR is calculated as *ω* = *q*/*r*_0_.

### Simulation algorithm

To study the time evolution of the GC reaction, we performed Brownian dynamics simulations. At every time point B cells proliferate or die with a probability which is determined by the time step and the proliferation and death rates (Table 2). We choose the time step to be smaller than the average time for proliferation. The simulation proceeds according to the following algorithm:

1. Initial seed of the GC with *M* clones.
2. **Growth phase**. B cells proliferate and die without competition according to a stochastic birth-death process with rates *β*_0_, *μ*_0_. This phase lasts *T*_*growth*_ days. The values of these parameters is detailed in Table 2.
3. **Competitive phase**. B cells proliferate and die with competition according to a stochastic birth-death process with birth rate $\beta_{i}=\beta_{0}\frac{C+A_{i}}{C+\langle A\rangle }$ (see Eq (1)) and logistic death rate $\mu (\text{n})=\left(\mu_{0}+\left(\beta_{0}-\mu_{0}\right)\frac{\sum_{i=1}^{M}n_{i}}{N}\right)$ (see Eq (6)). This phase lasts *T*_*comp*_ days. The values of these parameters is detailed in Table 2. In the stochastic simulation, at each time step:
  - B cells attempt to capture Ag (see section “Ag encounter and binding by the BCR”, “Ag internalization”).
  - B cells divide with the rate given by *β*_*i*_ (see Eq (1)). B cells have to capture at least 1 Ag molecule to attempt proliferation.
  - The progeny of a B cell mutates its BCR affinity (see section “Affinity change following BCR mutation”).
  - B cells die with a rate *μ*(**n**).

**Table 2:** Simulation parameters.

Parameters used in the simulation	Value
*M*: number of initial clones:	20
*μ*_0_: basal death rate	1 day^-1^
*β*_0_: basal birth rate	1.5 day^-1^
*N*: germinal center capacity	200
*D*: variability coefficient	0.05
*ω*_0_: initial affinity	1.5
*T*_*growth*_: Growth phase	4 days
*T*_*comp*_: competitive phase	16 days
*F*: Pulling force	1 pN
*E*_*Ag*−*mem*_: interaction energy between the Ag and the immune complex on the FDC	1k_B_T
*E*_*Ag*−*Ab*_: initial interaction energy between the Ag and the BCR. When mutating the bond energy this value change during the simulation.	0.4k_B_T
*q* (on-rate): initial activation rate for the encounter between the BCR and the Ag. When mutating the bond energy this value change during the simulation.	0.5 t.u^-1^
*x*_*b*_: the distance at which a bond breaks.	1nm
Number of BCR molecules per B cell	100
*τ*: the maximal search time of Ag by the BCR at the B cell protrusion in the immune synapse. After this time, the protrusion retracts with no Ag captured.	1 t.u
*p*: The probability that an Ag molecule will be in the searching radius of the BCR.	0.09
*C*: availability of T follicular help cells (TfhCs).	75

## Supporting information

S1 Text Spike densities of different viruses. (DOCX)

S2 Text This file provides a detailed explaination of S1 Fig showing that AM is optimal for intermediate spike density. (DOCX)

S3 Text Numerical methods. The file contains the details of the computations.
(DOCX)

S1 Fig Fraction of dominant clone, and the average affinity, for different simulation parameters. (**a-b**) The fraction of the GC occupied by the dominant clone (a) and its mean affinity (b) at day 16, where *E*_*Ag*−*Ab*_ changes upon mutation while *q* remains constant. Here the mutation probability is 0.5 while the other parameters are detailed in Table 2. **(c-d)** Simulations with different values of the basal birth rate. **(e-f)** Simulations with different population capacity *N*. **(g-h)** Simulations with different values of the variability coefficient D. **(i-j)** Simulation with different values of the death rate. (**k-l**) Simulation with BCR cluster of size 2. The variability coefficient taken here is D = 0.01, except for h. See details in S2 Text -“AM is largest for intermediate spike density”.
(EPS)

S2 Fig Selection and affinity maturation in a germinal center when the on-rate mutated and as a function of Ag encounter probability. (**a**) Mean affinity of the dominant clone as a function of Ag density. *q* changes upon mutation while *E*_*Ag*−*Ab*_ remains constant. (*p* = 0.09). The overall range of change is larger when mutating *E*_*Ag*−*Ab*_ (Fig 2a) because small changes in the energy following mutation are exponentiated (Eq (9)). (**b**) The fraction of the GC occupied by the dominant clone at day 16, where either *q* changes upon mutation while *E*_*Ag*−*Ab*_ remains constant (red), or vice versa (blue). (**c-d**) The BCR molecule does not diffuse freely in the synapse but performs confined stochastic motion, which depends on the interaction with the actin network [65]. Changing the search area of the BCR or its diffusion coefficient effectively changes the antigen encounter probability *p* (Eq (1)). Mean occupation fraction (c) and affinity (d) of the dominant clone as a function of the probability that the Ag is within the scanning radius of the BCR (*M*_*Ag*_ = 10). Each point on the curves was obtained by averaging over 400 independent GC reactions. The parameter that accounts for the availability of TfhCs was set to an intermediate value of *C* = 75. The variability coefficient taken here is D = 0.01.
(EPS)

S3 Fig Accumulated affinity of B cells. The mean affinity of a fraction of the B cells produces throughout the GCR. At each time point, we choose randomly 10% of the B cells in the GC. Their affinities were then averaged. The curve is a proxy for the affinities of memory and plasma B cells that would have been created during the GCR. The simulation parameters are detailed in Table 2.
(EPS)

S4 Fig Clonal diversity. (**a**) The fraction of the GC occupied by the dominant clone at day 16, where *E*_*Ag*−*Ab*_ changes upon mutation while *q* remains constant. The simulation parameters are detailed in Table 2. (**b**) The distribution of clonal dominance fraction for different GC realizations at days 1, 5, 10 and 16 of the GCR for *n*_*Ag*_ = 0.11.
(EPS)

S5 Fig Probability distribution of binding energy. The energy distribution evolution in time for *n*_*Ag*_ = 0.13.
(EPS)

S6 FigThe rate of affinity increase.The mean on-rate $\bar{q}$ and variance *σ*^2^ calculated using Eqs (S25) and (S26) at different times, the parameters *a* = 0.77, *b* = 0.38, *d*_1_ = 0.2, *d*_2_ = 0.2, and *D* = 0.05 match the parameters in Table 2 and the initial on-rate is ${\bar{q}}_{0}=0.01$. Each B cell has one BCR that has two arms. The time is in units of *τ*_0_. We solve the equations for *C*_1_ = 1 (a,b) and *C*_1_ = 0.25 (c,d).(EPS)

S7 FigThe rate of affinity increase for a BCR with one arm.**(a)** Scheme of a one arm BCR. Depicted are all the possible interaction states of the BCR and the Ag. (**b-e**) The mean on-rate calculated by solving numerically Eqs (S36,S39) at different times, the parameters *a* = 0.77, *b* = 0.38, *d*_1_ = 0.2, *d*_2_ = 0.2, and *D* = 0.05 that match the parameters in Table 2 while the initial on-rate is ${\bar{q}}_{0}=0.01$. The time is in units of *τ*_0_. We solve the equations for *C*_1_ = 1 with *τ* = 10(a), *τ* = 100(b) and *C*_1_ = 0.25 with *τ* = 10(c) and *τ* = 100(d).(EPS)

S8 FigMean affinity of B cells when the SD decreases with time.The affinity of B cells at day 16 of the GCR when the spike density decays exponentially as $n_{Ag}(t)=n_{Ag}(0)e^{-t/T_{Ag-decay}}$. The x-axis is the density at day zero of the competitive phase. Shown are the mean affinities when Ag does not decay (blue), *T*_*Ag*−*decay*_ = 16 days (yellow), and *T*_*Ag*−*decay*_ = 10 days (red).(EPS)

S9 Fig Dominance of clones following T helper cell restriction. The fraction of the dominant clone in a GC depending on the amount of available Tfh cells (*C*). Different curves correspond to different values of spike density (*n*_*Ag*_). *E*_*Ag*−*Ab*_ changes upon mutation in these simulations while *q* remains fixed.
(EPS)

S10 Fig The state of the BCR and the Ag. Illustrated are all the possible states of the BCR and the Ag molecules. The notation is explained in the methods section.
(EPS)

## References

[1] Bouvier NM, Palese P. The biology of influenza viruses. Vaccine. 2008;26[SUPPL. 4]:49–53. 10.1016/j.vaccine.2008.07.039 PMC3074182 19230160

[2] Mammen M, Choi S-K, Whitesides GM. Polyvalent Interactions in Biological Systems: Implications for Design and Use of Multivalent Ligands and Inhibitors. Angew Chemie Int Ed [Internet]. 1998;37[20]:2754–94. Available from: http://doi.wiley.com/10.1002/%28SICI%291521-3773%2819981102%2937%3A20%3C2754%3A%3AAID-ANIE2754%3E3.0.CO%3B2-3 10.1002/(SICI)1521-3773(19981102)37:20<2754::AID-ANIE2754>3.0.CO;2-3 29711117

[3] Galimidi RP, Klein JS, Politzer MS, Bai S, Seaman MS, Nussenzweig MC, et al Intra-spike crosslinking overcomes antibody evasion by HIV-1. Cell [Internet]. Elsevier Inc.; 2015;160[3]:433–46. Available from: 10.1016/j.cell.2015.01.016 25635457 PMC4401576

[4] Angeletti D, Gibbs JS, Angel M, Kosik I, Hickman HD, Frank GM, et al Defining B cell immunodominance to viruses. Nat Immunol [Internet]. 2017;18[4]:456–63. Available from: http://www.nature.com/doifinder/10.1038/ni.3680 28192417 10.1038/ni.3680 PMC5360521

[5] Yamaguchi M, Danev R, Nishiyama K, Sugawara K, Nagayama K. Zernike phase contrast electron microscopy of ice-embedded influenza A virus. J Struct Biol. 2008;162[2]:271–6. 10.1016/j.jsb.2008.01.009 18313941

[6] Merz A, Long G, Hiet M-S, Brügger B, Chlanda P, Andre P, et al Biochemical and morphological properties of hepatitis C virus particles and determination of their lipidome. J Biol Chem [Internet]. 2011;286[4]:3018–32. Available from: http://www.ncbi.nlm.nih.gov/pubmed/21056986%5Cnhttp://www.pubmedcentral.nih.gov/articlerender.fcgi?artid=PMC3024796 21056986 10.1074/jbc.M110.175018 PMC3024796

[7] Zhu P, Chertova E, Bess J, Lifson JD, Arthur LO, Liu J, et al Electron tomography analysis of envelope glycoprotein trimers on HIV and simian immunodeficiency virus virions. Proc Natl Acad Sci U S A [Internet]. 2003;100[26]:15812–7. Available from: http://www.ncbi.nlm.nih.gov/pubmed/14668432%5Cnhttp://www.pubmedcentral.nih.gov/articlerender.fcgi?artid=PMC307650 14668432 10.1073/pnas.2634931100 PMC307650

[8] Riedel C, Vasishtan D, Siebert CA, Whittle C, Lehmann MJ, Mothes W, et al Native structure of a retroviral envelope protein and its conformational change upon interaction with the target cell. J Struct Biol [Internet]. The Author(s); 2017;197[2]:172–80. Available from: 10.1016/j.jsb.2016.06.017 27345930 PMC5182179

[9] Forster F, Medalia O, Zauberman N, Baumeister W, Fass D. Retrovirus envelope protein complex structure in situ studied by cryo-electron tomography. Proc Natl Acad Sci [Internet]. 2005;102[13]:4729–34. Available from: http://www.pnas.org/cgi/doi/10.1073/pnas.0409178102 15774580 10.1073/pnas.0409178102 PMC555690

[10] Dolnik O, Volchkova V, Garten W, Carbonnelle C, Becker S, Kahnt J, et al Ectodomain shedding of the glycoprotein GP of Ebola virus. EMBO J. 2004;23[10]:2175–84. 10.1038/sj.emboj.7600219 15103332 PMC424403

[11] Stano A, Leaman DP, Kim AS, Zhang L, Autin L, Ingale J, et al Dense array of spikes on HIV-1 virion particles. J Virol [Internet]. 2017;[4]:JVI.00415-17. Available from: http://jvi.asm.org/lookup/doi/10.1128/JVI.00415-17 10.1128/JVI.00415-17 PMC5487557 28446665

[12] Victora GD, Nussenzweig MC. Germinal centers. Annu Rev Immunol [Internet]. 2012;30:429–57. Available from: http://www.ncbi.nlm.nih.gov/pubmed/22224772%5Cnhttp://www.annualreviews.org/doi/pdf/10.1146/annurev-immunol-020711-075032 22224772 10.1146/annurev-immunol-020711-075032

[13] Eisen HN, Siskind GW. Variations in Affinities of Antibodies During the Immune Response. Biochemistry [Internet]. 1964;3[1959]:996–1008. Available from: http://www.ncbi.nlm.nih.gov/pubmed/14214095 14214095 10.1021/bi00895a027

[14] Park CS, Choi YS. How do follicular dendritic cells interact intimately with B cells in the germinal centre? Immunology. 2005;114[1]:2–10. 10.1111/j.1365-2567.2004.02075.x 15606789 PMC1782056

[15] Carrasco YR, Batista FD. B Cells Acquire Particulate Antigen in a Macrophage-Rich Area at the Boundary between the Follicle and the Subcapsular Sinus of the Lymph Node. Immunity. 2007;27[1]:160–71. 10.1016/j.immuni.2007.06.007 17658276

[16] Rolf J, Bell SE, Kovesdi D, Michelle L, Soond DR, Webb LMC, et al Phosphoinositide 3-kinase activity in T cells regulates the magnitude of the germinal center reaction. J Immunol [Internet]. 2010;185[7]:4042–52. Available from: http://www.ncbi.nlm.nih.gov/pubmed/20826752 20826752 10.4049/jimmunol.1001730

[17] Gitlin AD, Mayer CT, Oliveira TY, Shulman Z, Jones MJK, Koren A, et al T cell help controls the speed of the cell cycle in germinal center B cells. Science (80-) [Internet]. 2015;349[July]:643–6. Available from: http://www.sciencemag.org/cgi/doi/10.1126/science.aac4919 10.1126/science.aac4919 PMC4809261 26184917

[18] Victora GD, Schwickert TA, Fooksman DR, Kamphorst AO, Meyer-Hermann M, Dustin ML, et al Germinal center dynamics revealed by multiphoton microscopy with a photoactivatable fluorescent reporter. Cell [Internet]. Elsevier Ltd; 2010;143[4]:592–605. Available from: 10.1016/j.cell.2010.10.032 21074050 PMC3035939

[19] Oprea M, Perelson AS. Somatic mutation leads to efficient affinity maturation when centrocytes recycle back to centroblasts. J Immunol [Internet]. 1997;158[11]:5155–62. Available from: http://www.ncbi.nlm.nih.gov/pubmed/9164931 9164931

[20] Kepler TB, Perelson aS. Cyclic re-entry of germinal center B cells and the efficiency of affinity maturation. Immunol Today. 1993;14[8]:412–5. 10.1016/0167-5699(93)90145-B 8397781

[21] Chaudhury S, Reifman J, Wallqvist A. Simulation of B cell affinity maturation explains enhanced antibody cross-reactivity induced by the polyvalent malaria vaccine AMA1. J Immunol. 2014;193[5]:2073–86. 10.4049/jimmunol.1401054 25080483 PMC4135178

[22] Wang S, Mata-Fink J, Kriegsman B, Hanson M, Irvine DJ, Eisen HN, et al Manipulating the selection forces during affinity maturation to generate cross-reactive HIV antibodies. Cell [Internet]. Elsevier Inc.; 2015;160[4]:785–97. Available from: 10.1016/j.cell.2015.01.027 25662010 PMC4357364

[23] Figge MT. Stochastic discrete event simulation of germinal center reactions. Phys Rev E—Stat Nonlinear, Soft Matter Phys. 2005;71[5]:1–15. 10.1103/PhysRevE.71.051907 16089571

[24] Zhang J, Shakhnovich EI. Optimality of mutation and selection in germinal centers. PLoS Comput Biol [Internet]. 2010;6[6]:e1000800 Available from: http://www.pubmedcentral.nih.gov/articlerender.fcgi?artid=2880589&tool=pmcentrez&rendertype=abstract 20532164 10.1371/journal.pcbi.1000800 PMC2880589

[25] Meyer-Hermann M, Mohr E, Pelletier N, Zhang Y, Victora GD, Toellner KM. A theory of germinal center b cell selection, division, and exit. Cell Rep. 2012;2[1]:162–74. 10.1016/j.celrep.2012.05.010 22840406

[26] Nowosad CR, Spillane KM, Tolar P. Germinal center B cells recognize antigen through a specialized immune synapse architecture. Nat Immunol [Internet]. Nature Publishing Group; 2016;17[May]:1–11. Available from: http://www.nature.com/doifinder/10.1038/ni.3458%5Cnhttp://www.ncbi.nlm.nih.gov/pubmed/27183103 27183103 10.1038/ni.3458 PMC4943528

[27] Tolar P, Spillane KM. Force generation in B-cell synapses: Mechanisms coupling B-cell receptor binding to antigen internalization and affinity discrimination [Internet]. 1st ed Advances in Immunology. Elsevier Inc; 2014 69–100 p. 10.1016/B978-0-12-800266-7.00002-9 24840948

[28] Jacob J, Przylepa J, Miller C, Kelsoe G. In situ studies of the primary immune response to (4-hydroxy-3-nitrophenyl)acetyl. III. The kinetics of V region mutation and selection in germinal center B cells. J Exp Med [Internet]. 1993;178[4]:1293–307. Available from: http://www.jem.org/cgi/doi/10.1084/jem.178.4.1293%5Cnhttp://www.pubmedcentral.nih.gov/articlerender.fcgi?artid=2191212&tool=pmcentrez&rendertype=abstract 8376935 10.1084/jem.178.4.1293 PMC2191212

[29] Gitlin AD, Shulman Z, Nussenzweig MC. Clonal selection in the germinal centre by regulated proliferation and hypermutation. Nature [Internet]. Nature Publishing Group; 2014;509[7502]:637–40. Available from: http://www.ncbi.nlm.nih.gov/pubmed/24805232 24805232 10.1038/nature13300 PMC4271732

[30] Shulman Z, Gitlin AD, Weinstein JS, Lainez B, Esplugues E, Flavell RA, et al Dynamic signaling by T follicular helper cells during germinal center B cell selection. Science (80-). 2014;345[6200]. 10.1126/science.1257861 PMC4519234 25170154

[31] Tas JMJ, Mesin L, Pasqual G, Targ S, Jacobsen JT, Mano YM, et al Visualizing antibody affinity maturation in germinal centers. Science (80-) [Internet]. 2016;58[12]:7250–7. Available from: http://www.ncbi.nlm.nih.gov/pubmed/25246403 10.1126/science.aad3439 PMC4938154 26912368

[32] Depoil D, Zaru R, Guiraud M, Chauveau A, Harriague J, Bismuth G, et al Immunological synapses are versatile structures enabling selective T cell polarization. Immunity. 2005;22[2]:185–94. 10.1016/j.immuni.2004.12.010 15723807

[33] de Vinuesa CG, Cook MC, Ball J, Drew M, Sunners Y, Cascalho M, et al Germinal centers without T cells. J Exp Med [Internet]. 2000;191[3]:485–94. Available from: http://www.ncbi.nlm.nih.gov/pubmed/10662794%5Cnhttp://www.pubmedcentral.nih.gov/articlerender.fcgi?artid=PMC2195827 10662794 10.1084/jem.191.3.485 PMC2195827

[34] Morgan HD, Dean W, Coker HA, Reik W, Petersen-Mahrt SK. Activation-induced cytidine deaminase deaminates 5-methylcytosine in DNA and is expressed in pluripotent tissues: Implications for epigenetic reprogramming. J Biol Chem. 2004;279[50]:52353–60. 10.1074/jbc.M407695200 15448152

[35] Amitai A, Mesin L, Victora GD, Kardar M, Chakraborty AK. A population dynamics model for clonal diversity in a germinal center. Front Microbiol. 2017;8[SEP]. 10.3389/fmicb.2017.01693 PMC5600966 28955307

[36] Fisher RA. The Genetical Theory of Natural Selection: A Complete Variorum Edition. Bennet JH, editor. Oxford, UK: Oxford University Press; 2000.

[37] Price GR. Selection and covariance. Nature. 1970;227[5257]:520–521. 5428476 10.1038/227520a0

[38] Okoye AA, Picker LJ. CD4+ T-Cell Depletion In Hiv Infection: Mechanisms Of Immunological Failure. Immunol Rev. 2013;254[1]:54–64. 10.1111/imr.12066 23772614 PMC3729334

[39] Satou Y, Matsuoka M. Virological and immunological mechanisms in the pathogenesis of human T-cell leukemia virus type 1. Rev Med Virol [Internet]. 2013 9 1 [cited 2017 Oct 24];23[5]:269–80. Available from: http://doi.wiley.com/10.1002/rmv.1745 23606621 10.1002/rmv.1745

[40] Motai Y, Takahashi M, Takachi T, Higuchi M, Hara T, Mizuguchi M, et al Human T-cell leukemia virus type 1 (HTLV-1) Tax1 oncoprotein but not HTLV-2 Tax2 induces the expression of OX40 ligand by interacting with p52/p100 and RelB. Virus Genes [Internet]. Springer US; 2016 2 6 [cited 2017 Oct 24];52[1]:4–13. Available from: http://link.springer.com/10.1007/s11262-015-1277-7 26739459 10.1007/s11262-015-1277-7

[41] Zingler K, Littman DR. Truncation of the cytoplasmic domain of the simian immunodeficiency virus envelope glycoprotein increases env incorporation into particles and fusogenicity and infectivity. J Virol [Internet]. 1993;67[5]:2824–31. Available from: http://www.ncbi.nlm.nih.gov/pubmed/8474176%5Cnhttp://www.pubmedcentral.nih.gov/articlerender.fcgi?artid=PMC237607 8474176 10.1128/jvi.67.5.2824-2831.1993 PMC237607

[42] Schiller J, Chackerian B. Why HIV Virions Have Low Numbers of Envelope Spikes: Implications for Vaccine Development. PLoS Pathog. 2014;10[8]:8–11. 10.1371/journal.ppat.1004254 PMC4125284 25101974

[43] Klein JS, Bjorkman PJ. Few and far between: How HIV may be evading antibody avidity. PLoS Pathog. 2010;6[5]:1–6. 10.1371/journal.ppat.1000908 PMC2877745 20523901

[44] Zhang Y, Meyer-Hermann M, George LA, Figge MT, Khan M, Goodall M, et al Germinal center B cells govern their own fate via antibody feedback. J Exp Med. 2013;210[3]:457–64. 23420879 10.1084/jem.20120150 PMC3600904

[45] McFarland HF. The effect of measles virus infection on T and B lymphocytes in the mouse. I. Suppression of helper cell activity. J Immunol [Internet]. 1974;113[6]:1978–83. Available from: http://www.ncbi.nlm.nih.gov/entrez/query.fcgi?cmd=Retrieve&db=PubMed&dopt=Citation&list_uids=4139203 4139203

[46] Auwaerter PG, Rota PA, Elkins WR, Adams RJ, DeLozier T, Shi Y, et al Measles virus infection in rhesus macaques: altered immune responses and comparison of the virulence of six different virus strains. J Infect Dis. 1999;180[4]:950–8. 10479117 10.1086/314993

[47] Griffin DE. The immune response in measles: Virus control, clearance and protective immunity. Viruses. 2016;8[10]. 10.3390/v8100282 PMC5086614 27754341

[48] Popovic M, Flomenberg N, Volkman DJ, Mann D, Fauci AS, Dupont B, et al Alteration of T-cell functions by infection with HTLV-I or HTLV-II. Science (80-) [Internet]. 1984;226[4673]:459–62. Available from: http://www.ncbi.nlm.nih.gov/entrez/query.fcgi?cmd=Retrieve&db=PubMed&dopt=Citation&list_uids=6093248 10.1126/science.6093248 6093248

[49] Igakura T, Stinchcombe JC, Goon PK, Taylor GP, Weber JN, Griffiths GM, et al Spread of HTLV-1 Between Lymphocytes by Virus-Induced Polarization of the Cytoskeletion. Science (80-) [Internet]. 2003;299[2003]:1713–6. Available from: http://science.sciencemag.org/content/299/5613/1713 10.1126/science.1080115 12589003

[50] Feuer G, Green PL. Comparative biology of human T-cell lymphotropic virus type 1 (HTLV-1) and HTLV-2. Oncogene. 2005;24[39]:5996–6004. 10.1038/sj.onc.1208971 16155606 PMC2659530

[51] Cao S, Maldonado JO, Grigsby IF, Mansky LM, Zhang W. Analysis of human T-cell leukemia virus type 1 particles by using cryo-electron tomography. J Virol [Internet]. 2015;89[4]:2430–5. Available from: http://www.scopus.com/inward/record.url?eid=2-s2.0-84921632829&partnerID=tZOtx3y1 25473052 10.1128/JVI.02358-14 PMC4338869

[52] Crooks ET, Jiang P, Franti M, Wong S, Zwick MB, Hoxie JA, et al Relationship of HIV-1 and SIV envelope glycoprotein trimer occupation and neutralization. Virology. 2008;377[2]:364–78. 10.1016/j.virol.2008.04.045 18539308 PMC2516379

[53] Edgeworth J, Freemont P, Hogg N. siv adaptation to human cells. Nature. 1989;342:189–92. 2478889 10.1038/342189a0

[54] Chakrabarti L, Emerman M, Tiollais P, Sonigo P. The cytoplasmic domain of simian immunodeficiency virus transmembrane protein modulates infectivity. J Virol. 1989;63[10]:4395–403. 2778881 10.1128/jvi.63.10.4395-4403.1989 PMC251057

[55] Kodama T, Wooley DP, Naidu YM, Kestler HW, Daniel MD, Li Y, et al Significance of premature stop codons in env of simian immunodeficiency virus. J Virol [Internet]. 1989;63[11]:4709–14. Available from: http://www.pubmedcentral.nih.gov/articlerender.fcgi?artid=251107&tool=pmcentrez&rendertype=abstract 2795718 10.1128/jvi.63.11.4709-4714.1989 PMC251107

[56] Dintzis HM, Dintzis RZ, Vogelstein B. Molecular determinants of immunogenicity: the immunon model of immune response. Proc Natl Acad Sci U S A. 1976;73[10]:3671–5. 62364 10.1073/pnas.73.10.3671 PMC431180

[57] Ingale J, Stano A, Guenaga J, Sharma SK, Nemazee D, Zwick MB, et al High-Density Array of Well-Ordered HIV-1 Spikes on Synthetic Liposomal Nanoparticles Efficiently Activate B Cells. Cell Rep [Internet]. The Author(s); 2016;15[9]:1986–99. Available from: 10.1016/j.celrep.2016.04.078 27210756 PMC4889521

[58] Hadzhieva M, Pashov AD, Kaveri S, Lacroix-Desmazes S, Mouquet H, Dimitrov JD. Impact of Antigen Density on the Binding Mechanism of IgG Antibodies. Sci Rep [Internet]. 2017;7[1]:3767 Available from: http://www.nature.com/articles/s41598-017-03942-z 10.1038/s41598-017-03942-z PMC5476644 28630473

[59] Nåsell I. Extinction and quasi-stationarity in the Verhulst logistic model. J Theor Biol [Internet]. 2001;211[1]:11–27. Available from: http://www.ncbi.nlm.nih.gov/pubmed/11407888 11407888 10.1006/jtbi.2001.2328

[60] Bell G. Models for the specific adhesion of cells to cells. Science (80-) [Internet]. 1978;200[4342]:618–27. Available from: http://www.sciencemag.org/cgi/doi/10.1126/science.347575 10.1126/science.347575 347575

[61] Schuss Z. Diffusion and Stochastic Processes An Analytical Approach. New York, NY: Springer-Verlag; 2009.

[62] Gru K, Desai P, Winkler DC, Heymann JB, Belnap DM, Baumeister W, et al Three-Dimensional Structure of Herpes Simplex Virus from Cryo—Electron Tomography. Science (80-). 2003;302[November]:1396–8. 10.1126/science.1090284 14631040

[63] Zhang X, Sheng J, Austin SK, Hoornweg TE, Smit JM, Kuhn RJ, et al Structure of acidic pH dengue virus showing the fusogenic glycoprotein trimers. J Virol [Internet]. 2015;89[1]:743–50. Available from: http://www.ncbi.nlm.nih.gov/pubmed/25355881%5Cnhttp://www.pubmedcentral.nih.gov/articlerender.fcgi?artid=PMC4301137 25355881 10.1128/JVI.02411-14 PMC4301137

[64] Prasad VM, Miller AS, Klose T, Sirohi D, Buda G, Jiang W, et al Structure of the immature Zika virus at 9 Å resolution. Nat Struct Mol Biol [Internet]. Nature Publishing Group; 2017;24[2]:184–6. Available from: http://www.nature.com/doifinder/10.1038/nsmb.3352 28067914 10.1038/nsmb.3352 PMC5296287

[65] Batista FD, Treanor B, Harwood NE. Visualizing a role for the actin cytoskeleton in the regulation of B-cell activation. Immunol Rev. 2010;237[1]:191–204. 20727037 10.1111/j.1600-065X.2010.00943.x

