# Supplementary material for: The low spike density of HIV may have evolved because of the effects of T helper cell depletion on affinity maturation
Source: PLoS Comput Biol. 2018 Aug 30;14(8):e1006408. doi: 10.1371/journal.pcbi.1006408 (PMC6150518; doi:10.1371/journal.pcbi.1006408)
Supplement: S1 Text — (DOCX) [file pcbi.1006408.s001.docx]

### Spike density of different viruses

In Table 1 we list the surface spike density of a number of viruses. Interestingly, for all viruses for which we could find data, the density is of the order of 1 spike per 100 nm^2^. We show that low or high Ag density can lead to ineffective B cell selection. Some viruses have large variability in their spike number, such as the respiratory syncytial virus (RSV), that produces virions with either very high or low spike density [1]. Similarly, EM images of the HCV lipoviral particles show that it can have high glycoprotein spike density on one side, and no spikes at all on the other side [2]. HIV however, has a very low spike density of 0.01 spikes per 100 nm^2^, which is two orders of magnitude smaller than that of other viruses. As far as we could find, there are no viruses with an intermediate spike density. While low spike density can prolong the development of good Abs, as we have shown, it also reduces the virus infectivity [3]. It is not clear if HIV infectivity of CD4+ is significantly hampered by the low spikes density [4]. The spike is mobile in the virus envelope and can form gp120 clusters when in contact with the T cell, assisting in entry [5].

### References

1. Liljeroos L, Krzyzaniak MA, Helenius A, Butcher SJ. Architecture of respiratory syncytial virus revealed by electron cryotomography. Proc Natl Acad Sci U S A [Internet]. 2013;110[27]:11133–8. Available from: http://www.ncbi.nlm.nih.gov/pubmed/23776214%5Cnhttp://www.pubmedcentral.nih.gov/articlerender.fcgi?artid=PMC3703984%5Cn%3CGo to ISI%3E://000321978000062

2. Piver E, Boyer A, Gaillard J, Bull A, Beaumont E, Roingeard P, et al. Ultrastructural organisation of HCV from the bloodstream of infected patients revealed by electron microscopy after specific immunocapture. Gut [Internet]. 2016;[October]:gutjnl-2016-311726. Available from: http://gut.bmj.com/lookup/doi/10.1136/gutjnl-2016-311726

3. Zingler K, Littman DR. Truncation of the cytoplasmic domain of the simian immunodeficiency virus envelope glycoprotein increases env incorporation into particles and fusogenicity and infectivity. J Virol [Internet]. 1993;67[5]:2824–31. Available from: http://www.ncbi.nlm.nih.gov/pubmed/8474176%5Cnhttp://www.pubmedcentral.nih.gov/articlerender.fcgi?artid=PMC237607

4. Magnus C, Rusert P, Bonhoeffer S, Trkola A, Regoes RR. Estimating the stoichiometry of human immunodeficiency virus entry. J Virol. 2009;83[3]:1523–31.

5. Sougrat R, Bartesaghi A, Lifson JD, Bennett AE, Bess JW, Zabransky DJ, et al. Electron tomography of the contact between T cells and SIV/HIV-1: Implications for viral entry. PLoS Pathog. 2007;3[5]:0571–81.
