# Supplementary material for: The low spike density of HIV may have evolved because of the effects of T helper cell depletion on affinity maturation
Source: PLoS Comput Biol. 2018 Aug 30;14(8):e1006408. doi: 10.1371/journal.pcbi.1006408 (PMC6150518; doi:10.1371/journal.pcbi.1006408)
Supplement: S2 Text — (DOCX) [file pcbi.1006408.s002.docx]

### AM is largest for intermediate spike density regardless of the model parameters

We verified that the qualitative behavior of the selection of the dominant clone, and the non-monotonic affinity curve, do not depend on our specific choice of parameters, but persist when decreasing the mutation probability (S 1a,b Fig), changing the basal birth-rate (S1c,d Fig), population capacity (S1e,f Fig), variability coefficient (S1g,h Fig) and death rate (S1i,j Fig). We also show simulation results when the BCR forms clusters (S1k,l Fig). In this case, there are 50 clusters, each containing 2 BCRs. Each cluster can bind between zero and four Ag molecules.
