# Supplementary material for: The low spike density of HIV may have evolved because of the effects of T helper cell depletion on affinity maturation
Source: PLoS Comput Biol. 2018 Aug 30;14(8):e1006408. doi: 10.1371/journal.pcbi.1006408 (PMC6150518; doi:10.1371/journal.pcbi.1006408)
Supplement: S3 Text — The file contains the details of the computations. (DOCX) [file pcbi.1006408.s003.docx]

## Numerical methods

### Estimating the Ag capture probabilities

To calculate the result of a capture attempt, we define the capture process of Ag by the BCR as an absorbing Markov chain and estimate the probabilities of the different outcomes (Figure 1). We describe the process with four bits. The first bit corresponds to the bound state of the first arm, the second to the second arm, the third the bond between one Ag molecule with the IC, or the virus membrane in our case, and the fourth of a second Ag molecule with the IC. Thus, 1111 is a state where both arms are connected to the Ags and both Ags are attached to the IC. 0111 is a state where only one arm is connected to an Ag molecule, while both Ags are attached to the IC (Figure 1ciii), 0011 is a state where none of the arms are connected to the Ags, while they are attached to the IC, 1100 is a state where both arms are connected to the Ags and they have detached from the IC, etc. All states are illustrated in S10 Fig. We define the transition probabilities between all states as:

$$\begin{matrix} \text{Transition states} \\ 1111\to\left\{\begin{matrix} 0111 & \text{w.p.} a_{1} \\ 1011 & \text{w.p.} a_{1} \\ 1101 & \text{w.p.} a_{2} \\ 1110 & \text{w.p.} a_{2} \end{matrix}\right. \\ 1110\to\left\{\begin{matrix} 1111 & \text{w.p.} c_{1} \\ 0110 & \text{w.p.} c_{2} \\ 1100 & \text{w.p.} c_{3} \end{matrix}\right. \\ 1101\to\left\{\begin{matrix} 1111 & \text{w.p.} c_{1} \\ 1001 & \text{w.p.} c_{2} \\ 1100 & \text{w.p.} c_{3} \end{matrix}\right. \\ 1011\to\left\{\begin{matrix} 1111 & \text{w.p.} b_{1} \\ 0011 & \text{w.p.} b_{2} \\ 1001 & \text{w.p.} b_{3} \end{matrix}\right. \\ 0111\to\left\{\begin{matrix} 1111 & \text{w.p.} b_{1} \\ 0011 & \text{w.p.} b_{2} \\ 0110 & \text{w.p.} b_{3} \end{matrix}\right. \end{matrix}$$

where the transition probabilities are

$a_{1}=\frac{k}{2k+2\xi} ; a_{2}=\frac{\xi}{2k+2\xi}$ ,

,

.

Here, ν is the on-rate for the Ag and membrane, ξ is the off-rate of the Ag from the membrane when two arms are bound and λ is the off-rate when one arm is bound. If we take $\lambda_{0}=e^{-\beta E_{Ag-mem}}$ to be the off-rate when no force is applied, the two off-rates are

$$\begin{matrix} \xi=\lambda_{0}e^{\beta x_{b}F/2}, \\ \lambda=\lambda_{0}e^{\beta x_{b}F}. \end{matrix}$$

$k$ is the arm rupture rate when two arms are bound and $r$ is the arm rupture rate when one arm is bound

where $r_{0}=e^{-\beta E_{Ag-Ab}}$. $q$ is the basal on-rate. Since there are a total of $N_{Ag}$ of Ag molecules in the IC with which the BCR can potentially interact, and one arm is bound to an Ag, there are $N_{Ag}-1$ free Ag molecules.

When only one Ag molecule is present

$$\begin{matrix} 0101\to\left\{\begin{matrix} 0100 & \text{w.p.} e_{1}, \\ 0001 & \text{w.p.} e_{2}, \end{matrix}\right. \\ 1010\to\left\{\begin{matrix} 0010 & \text{w.p.} e_{1}, \\ 1000 & \text{w.p.} e_{2}, \end{matrix}\right. \end{matrix}$$

where

$e_{1}=\frac{\lambda}{r+\lambda} ; e_{2}=\frac{r}{r+\lambda}$ .

Since at least one Ag molecule should be present for the capture process to start, the state 0000 does not partake.

The absorbing states when at least 2 Ag molecules are present are: 1100,1001,0110,0011. When only one Ag is present the absorbing states are: 0100,0001,0010,1000.

By solving the absorbing Markov chain, we find the capture and rupture probabilities, given that at the beginning of the process one of the arms was bound to an Ag molecule. When two or more Ag molecules are present ($N_{Ag}>1$), the possible initial states are 1011 or 0111 and the probabilities are

$$\begin{matrix} P_{\text{Capture;2Ag}}=\frac{q\left(N_{Ag}-1\right)\lambda\xi}{\left(r+\lambda\right)\left(k\left(r+\lambda+\nu\right)+\left(q\left(N_{Ag}-1\right)+r+\lambda\right)\xi\right)}, \\ P_{\text{Capture;1Ag}}=\frac{k\lambda\left(r+\lambda+\nu\right)+\left(q\left(N_{Ag}-1\right)r+\lambda\left(r+\lambda\right)\right)\xi}{\left(r+\lambda\right)\left(k\left(r+\lambda+\nu\right)+\left(q\left(N_{Ag}-1\right)+r+\lambda\right)\xi\right)}, \\ P_{\text{Rupture}}=\frac{r\left(k\left(r+\lambda+\nu\right)+\left(r+\lambda\right)\xi\right)}{\left(r+\lambda\right)\left(k\left(r+\lambda+\nu\right)+\left(q\left(N_{Ag}-1\right)+r+\lambda\right)\xi\right)}. \end{matrix}$$

When only one Ag molecule is present ($N_{Ag}=1$) the capture/rupture probabilities are

$$\begin{matrix} P_{\text{Capture;1Ag}}=e_{1}, \\ P_{\text{Rapture}}=e_{2}. \end{matrix}$$

### The probability of weaker affinity B cell selection

$P_{\text{weaker aff selection}}$ is the probability that in any given round of mutation and selection, a B cell with a lower affinity for the antigen gets selected and expands in favor of another B cell that has a higher affinity. The probability of a lower affinity cell dividing before a higher affinity one is

$P_{\text{weaker aff selection}}=\frac{\beta_{1}}{\beta_{1}+\beta_{2}}$,

where $\beta_{1}$ and $\beta_{2}$ are respectively the birthrates of cells 1 and 2 (equation ), and we assume that the antigen binding affinity of cell 1 is smaller than that of cell 2 ($\omega_{1}<\omega_{2}$).

The single BCR Ag capture probabilities are (see Figure 2c)

$$\begin{matrix} p\left(A=0\right)=p_{0}+p_{1}P_{\text{Rupture}}, \\ p\left(A=1\right)=p_{1}P_{\text{Capture;1Ag}}, \\ p\left(A=2\right)=p_{1}P_{\text{Capture;2Ag}}, \end{matrix}$$

with $p_{0}$ (respectively$p_{1}$) is the probability of finding (respectively not finding) an Ag molecule during a time $\tau$ (equation ) before the protrusion on the B cell retracts (see Figure 1c), $P_{\text{Rapture}}$,$P_{\text{Capture;1Ag}}$ and $P_{\text{Capture;2Ag}}$ are the extraction probabilities, starting with one arm of the BCR bound to an Ag molecule (see equation ).

Since a cell has 100 BCRs, it can capture between 0 and 200 Ags. The probability of cell 1 to proliferate before cell 2 given that it has a smaller affinity is

$$\begin{matrix} P_{\text{weaker aff selection}}=\int_{\omega_{1}} d\omega_{1}\int_{\omega_{1}<\omega_{2}} d\omega_{2}P\left(\omega_{1},\omega_{2}\right)\sum_{\begin{matrix} i,j=0 \\ i>0 \text{or }j>0 \end{matrix}}^{200} \frac{i+C}{i+j+2C}p\left(A_{1}=i,\omega_{1};A_{2}=j,\omega_{2}|i>0 \text{or j}>0\right)= \\ \int_{\omega_{1}} d\omega_{1}\int_{\omega_{1}<\omega_{2}} d\omega_{2}P\left(\omega_{1},\omega_{2}\right)\sum_{\begin{matrix} i,j=0 \\ i>0 \text{or }j>0 \end{matrix}}^{200} \frac{i+C}{i+j+2C}\frac{p\left(A_{1}=i,\omega_{1}\right)p\left(A_{2}=j,\omega_{2}\right)}{p\left(i>0 \text{or j}>0\right)}, \end{matrix}$$

where$p\left(A_{1}=i,\omega_{1}\right)$ is the probability of a B cell capturing $i$ Ag molecules given affinity $\omega_{1}$, $P\left(\omega_{1},\omega_{2}\right)$ is the joint distribution of the affinities for cell 1 and 2, and a B cell can only proliferate if it captures at least one Ag molecule.

### Estimating the rate of affinity increase

We compute here the rate of mean affinity increase of the B cell population. Our goal is to find the scaling of the optimal density (for which affinity increase is largest) with the model parameters and the result will not be directly compared to our affinity curve (Figure 2a). For the following calculations, we consider that each B cell has only a single BCR. We assume that only the on-rate changes upon mutation and that the change is drawn from a normal distribution (equation )

$\frac{dq}{dt}=\sqrt{2D}\frac{dB}{dt}$,

where $\frac{dB}{dt}$ is a white Gaussian noise with mean zero and variance 1.

We further assume that the time allowed to find the first Ag molecule is longer than the effective on-rate ($qn\ll\tau$), where $n=M_{Ag}p$ (see “Model description”) is the Ag density which we take to be a continuous variable. Thus, $p_{0}=0,p_{1}=1$ (see equation ) when $n>0$. The single BCR capture probabilities (equation ) are thus of the form

$$\begin{matrix} h_{0}=\frac{a}{a+b+(d_{1}+d_{2})qn}, \\ h_{1}=\frac{b+d_{1}qn}{a+b+(d_{1}+d_{2})qn}, \\ h_{2}=\frac{d_{2}qn}{a+b+(d_{1}+d_{2})qn}, \end{matrix}$$

where $a=rk(r+\lambda+\upsilon)+r\xi(r+\lambda)$, $b=\lambda k(r+\lambda+\upsilon)+\lambda\xi(r+\lambda)$, $d_{1}=r\xi$, $d_{2}=\lambda\xi$ (see equation ), $q$ the on-rate.

Let $x_{i,q}$ be the fraction of cells that belong to clone $i$ and have on-rate $q$, such that $\int\sum_{i} x_{i,q}dq=\int p(q)dq=1$, where $p(q)$ is the population distribution of $q$. The fractions evolve with time according to replicator equation

$\tau_{0}\frac{dx_{i,q}}{dt}=x_{i,q}\left(f_{q}-\left\langle f\right\rangle\right)$,

where $\tau_{0}$ is the rate of population growth determined by the basal birth rate and death rate. The fitness (Ag-dependent birthrate) $f_{q}$ is given by

$f_{q}=\frac{C_{1}+A_{q}}{C_{1}+\left\langle A\right\rangle}$,

where $A_{q}$ is a random number which is the amount of Ag captured by a BCR with on-rate $q$, $C_{1}$ is the parameter describing the availability of TfhCs, given that each B cell has only a single BCR, and $\left\langle A\right\rangle$ is the average amount of Ag captured by the population. The mean fitness of the population is $\langle f\rangle=1$.

The mean on-rate $\bar{q}$ is given by

$\bar{q}=\int q\sum_{i} \left\langle x_{i,q}\right\rangle dq$.

The average of the clonal fraction $i$ with on-rate $q$ ($\left\langle x_{i,q}\right\rangle$) is over the different realizations of the stochastic selection process. We now find the time evolution of the first and second moments of the average distribution on-rate distribution $\bar{p}(q)=\sum_{i} \left\langle x_{i,q}\right\rangle$.

$\bar{q}$ evolves with time as

$\frac{d\bar{q}}{dt}=\int\sum_{i} \left(\frac{d\left\langle x_{i,q}\right\rangle}{dt}q+\left\langle x_{i,q}\right\rangle\frac{dq}{dt}\right)dq=\tau_{0}^{-1}\frac{\mathrm{cov}(q,A)}{\left\langle A\right\rangle+C_{1}}$,

where we used $\left\langle\frac{dq}{dt}\right\rangle=0$. To solve equation , we first find an approximation for the covariance of $q$ and $A$. The covariance is approximated by

$$\mathrm{cov}(q,A)=\sum_{j=0}^{2} \int q\bar{p}(q)jh_{j}(q)dq-\bar{q}\left\langle A\right\rangle\approx\sigma^{2}A_{1,\bar{q}},$$

where $\sigma^{2}$ is the variance of $\bar{p}(q)$ and we performed a Taylor expansion around $\bar{q}$ to the second order: $A_{1,\bar{q}}\equiv\sum_{j=0}^{2} j\left.\frac{\partial h_{j}}{\partial q}\right|_{\bar{q}}$and $A_{2,\bar{q}}=\sum_{j=0}^{2} j\left.\frac{\partial^{2}h_{j}}{\partial q^{2}}\right|_{\bar{q}}$. The approximation is only valid when the amount of capture Ag $A_{q}$ is smaller than the asymptotic amount of captured Ag for large $q$. Towards the asymptotic value, the first order Taylor expansion is zero, and high order moments contribute. Substituting equation to equation we find

$\tau_{0}\frac{d\bar{q}}{dt}\simeq\sigma^{2}\frac{A_{1,\bar{q}}}{\left\langle A\right\rangle+C_{1}}\simeq\sigma^{2}\frac{A_{1,\bar{q}}}{A_{\bar{q}}+C_{1}}$.

We now find the time evolution of $\sigma^{2}$:

$$\frac{d\sigma^{2}}{dt}=\int\sum_{i} \frac{d\left\langle x_{i,q}\right\rangle}{dt}\left(q-\bar{q}\right)^{2}dq+\int\sum_{i} \left\langle x_{i,q}\right\rangle\frac{d\left(q-\bar{q}\right)^{2}}{dt}dq.$$

Since the on-rate mutates according to equation , using Ito’s formula [1] we find

$\frac{d\left(q-\bar{q}\right)^{2}}{dt}=2D+2\left(q-\bar{q}\right)\frac{dB_{t}}{dt}$.

The first term on the right-hand side of equation is

$$\int\sum_{i} \frac{d\left\langle x_{i,q}\right\rangle}{dt}\left(q-\bar{q}\right)^{2}dq=\tau_{0}^{-1}\frac{\mathrm{cov}\left(\left(q-\bar{q}\right)^{2},A\right)}{\left\langle A\right\rangle+C_{1}},$$

where we used $\left\langle x_{i,q}A{}_{q}\right\rangle=\left\langle x_{i,q}\right\rangle\left\langle A{}_{q}\right\rangle$ because the result of a capture event of a BCR with on-rate $q$ at time $t$ does not depend on the distribution of on-rates in the population at the same time.

Substituting equations and back to equation , and averaging, we find

$$\tau_{0}\frac{d\sigma^{2}}{dt}=\frac{\mathrm{cov}\left(\left(q-\bar{q}\right)^{2},A\right)}{\left\langle A\right\rangle+C_{1}}+2D.$$

Similarly to the calculation of the time evolution of the first moment, the covariance can be approximated by expanding $A_{q}$ around the mean on-rate

$$\mathrm{cov}\left(\left(q-\bar{q}\right)^{2},A\right)\simeq A_{1,\bar{q}}\left\langle\left(q-\bar{q}\right)^{3}\right\rangle+\frac{1}{2}A_{2,\bar{q}}\left\langle\left(q-\bar{q}\right)^{4}\right\rangle-\frac{1}{2}A_{2,\bar{q}}\left\langle\left(q-\bar{q}\right)^{2}\right\rangle^{2},$$

where $\left\langle\left(q-\bar{q}\right)^{3}\right\rangle,\left\langle\left(q-\bar{q}\right)^{4}\right\rangle$ are the 3^rd^ and 4^th^ central moments of the distribution $\bar{p}(q)$.

The higher order moments of the distribution of variation (mutations) in $q$ are zero (normal distribution - equation ). Thus, the evolution of the 3^rd^ central moment is

$\tau_{0}\frac{d\left\langle\left(q-\bar{q}\right)^{3}\right\rangle}{dt}=\frac{\mathrm{cov}\left(\left(q-\bar{q}\right)^{3},A\right)}{\left\langle A\right\rangle+C_{1}}\simeq\frac{\left\langle\left(q-\bar{q}\right)^{4}\right\rangle}{\left\langle A\right\rangle+C_{1}}A_{1,\bar{q}}$.

At the starting point of our GC simulation, all B cells have the same affinity. Thus, the affinity distribution is a delta function. As a result, the kurtosis $K=\left\langle\left(q-\bar{q}\right)^{4}\right\rangle/\sigma^{4}$ and skewness $S=\left\langle\left(q-\bar{q}\right)^{3}\right\rangle/\sigma^{3}$ are both zero at the beginning of the competitive phase. During the competitive phase, at any time $t$, the kurtosis and skewness are related by $K_{t}\sim3+\frac{3}{2}S_{t}^{2}$ [2]. The former relation is correct since half of the changes in $q$ following mutation are beneficial (see [2] theorem 3). Thus, during the GCR both $K_{t}$ and $S_{t}$ are positive. Additionally, $\bar{p}(q)$ converges to a normal distribution as $t$ grows. Thus, $S_{t}$ converge to zero for large $t$. For these reasons, we approximate the 3^rd^ central moment as zero. Assuming that $\bar{p}(q)$ is normal $\left\langle\left(q-\bar{q}\right)^{4}\right\rangle=3\sigma^{4}$. Thus

$\mathrm{cov}\left(\left(q-\bar{q}\right)^{2},A\right)\simeq A_{2,\bar{q}}\sigma^{4}$.

We now find the explicit equations for $d\bar{q}/dt$ and $d\sigma^{2}/dt$.The zero, first and second order expansion coefficients of $A_{q}$ around $\bar{q}$ are

$$\begin{matrix} A_{\bar{q}}=\frac{b+\left(d_{1}+2d_{2}\right)\bar{q}n}{\left(a+b+\left(d_{1}+d_{2}\right)\bar{q}n\right)}, \\ A_{1,\bar{q}}=\frac{\left(bd_{2}+a(d_{1}+2d_{2}\right)n}{\left(a+b+\left(d_{1}+d_{2}\right)\bar{q}n\right)^{2}}, \\ A_{2,\bar{q}}=-\frac{2n^{2}\left(d_{1}+d_{2}\right)\left(ad_{1}+\left(2a+b\right)d_{2}\right)}{\left(a+b+n\bar{q}(d_{1}+d_{2})\right)^{3}}. \end{matrix}$$

Substituting equation into equation

.

Substituting equations , into equation , we find

$\tau_{0}\frac{d\sigma^{2}}{dt}\simeq2D-\frac{2n^{2}\left(d_{1}+d_{2}\right)\left(ad_{1}+\left(2a+b\right)d_{2}\right)}{\left(\frac{b+\left(d_{1}+2d_{2}\right)\bar{q}n}{\left(a+b+\left(d_{1}+d_{2}\right)\bar{q}n\right)}+C_{1}\right)\left(a+b+n\bar{q}(d_{1}+d_{2})\right)^{3}}\sigma^{4}$.

Thus, the variance and mean on-rate of the distribution evolve according to two coupled nonlinear equations.

We solve equations , numerically and plot the solution in S6 Fig. Interestingly, the solution exhibits the same non-monotonous behavior as in the GCR simulation (S2a Fig) and when TfhCs are abundant (larger $C_{1}$), the increase in affinity is more moderate (S2b Fig).

Finally, we find that the optimal density (for which affinity increase is largest) at a specific mean on-rate and a given variance, for which the rate of increase in affinity is maximal is

$n^{*}=\frac{\left(r+\lambda\right)\left(k+\xi\right)}{\bar{q}\xi}\sqrt{\frac{\lambda+C_{1}\left(r+\lambda\right)}{\left(1+C_{1}\right)r+\left(2+C_{1}\right)\lambda}}$,

where $\lambda$ and $r$ are bonds rupture rates (see the previous section in SI). When the interaction energy of the Ab and the Ag is equal to that of the Ag with the IC ($E_{Ag-Ab}=E_{Ag-mem}$), $k=\xi$ and $r=\lambda$, the optimal density is

$n^{*}=4\frac{\lambda_{0}}{\bar{q}}e^{\beta x_{b}F}\sqrt{\frac{1+2C_{1}}{3+2C_{1}}}$.

When the interaction energy of the Ab and the Ag is much larger than that of the Ag with the IC ($E_{Ag-Ab}\gg E_{Ag-mem}$), $k\ll\xi$ and $r\ll\lambda$, the optimal density is

$n^{*}=\frac{\lambda_{0}}{\bar{q}}e^{\beta x_{b}F}\sqrt{\frac{1+C_{1}}{2+C_{1}}}$.

### Asymptotic behavior of the mean on-rate equation

The above result is only valid when the amount of captured Ag $A_{q}$ is smaller than its asymptotic value for large $q$. For large on-rate ($q\gg\frac{b}{n(d_{1}+d_{2})}$), the amount of captured Ag is $A_{q}\simeq\frac{d_{1}+2d_{2}}{d_{1}+d_{2}}$. To study the behavior in this limit, we expand $A(q)$ around infinity

,

where

$$\begin{matrix} A_{\infty,0}=\frac{d_{1}+2d_{2}}{d_{1}+d_{2}}, \\ A_{\infty,1}=\frac{ad_{1}+2ad_{2}+bd_{2}}{\left(d_{1}+d_{2}\right)^{2}}, \\ A_{\infty,2}=\frac{a^{2}d_{1}+abd_{1}+2a^{2}d_{2}+3abd_{2}+b^{2}d_{2}}{\left(d_{1}+d_{2}\right)^{3}}. \end{matrix}$$

The covariance is approximated by

$$\mathrm{cov}(q,A)\simeq n^{-1}\left[\left\langle q_{n}\right\rangle A_{\infty,0}-A_{\infty,1}+A_{\infty,2}\left\langle\frac{1}{q_{n}}\right\rangle-\left\langle q_{n}\right\rangle\left(A_{\infty,0}-A_{\infty,1}\frac{1}{\left\langle q_{n}\right\rangle}+A_{\infty,2}\frac{1}{\left\langle q_{n}\right\rangle^{2}}\right)\right]\simeq0,$$

where $q_{n}\equiv qn$ and we used

$$\left\langle\frac{1}{q_{n}}\right\rangle\simeq\frac{1}{\left\langle q_{n}\right\rangle} ; \left\langle\frac{1}{q_{n}^{2}}\right\rangle\simeq\frac{1}{\left\langle q_{n}\right\rangle^{2}}.$$

Thus, for large $q_{n}$

$\tau_{0}\frac{d\bar{q}}{dt}=\frac{\mathrm{cov}(q,A)}{\left\langle A\right\rangle+C_{1}}\to0$.

The mean on-rate will stop increasing as $d\bar{q}/dt\to0$ and will reach an asymptotic value. Since $\bar{q}$ reaches an asymptotic value, so does the negative coefficient $\frac{A_{2,\bar{q}}}{\left\langle A\right\rangle+C_{1}}$ in front of the second term in the right-hand side of equation . This term serves as a “stopping” force as the variance increases. Thus, the variance also reaches an asymptotic value.

### Estimating the rate of affinity increase when the Ab has only one arm

We now find the rate of increase in the mean affinity in a scenario where the Ab has only one arm (see S7a Fig). While in the two arms case we assumed the time to find the first Ag molecule was very long ($qn\ll\tau$), when the BCR has only one arm, the capture probability depends on Ag density only through the initial search. Thus, we do not take the limit of large $\tau$, and the capture probabilities in this case are

where the initial search probabilities are given by equation . Similarly to the two arms case

$\tau_{0}\frac{d\bar{q}}{dt}=\sigma^{2}\frac{A_{1,\bar{q}}}{C_{1}+\left\langle A\right\rangle}$.

Using the Taylor expansion of $A_{q}$

$$\begin{matrix} A_{\bar{q}}=\frac{\lambda}{k+\lambda}\left(1-e^{-\bar{q}n\tau}\right), \\ A_{1,\bar{q}}=\sum_{j=0}^{1} j\left.\frac{\partial h_{j}}{\partial q}\right|_{\bar{q}}=\frac{\lambda}{k+\lambda}n\tau e^{-\bar{q}n\tau}, \\ A_{2,\bar{q}}=\sum_{j=0}^{1} j\left.\frac{\partial^{2}h_{j}}{\partial q^{2}}\right|_{\bar{q}}=-\frac{\lambda}{k+\lambda}\left(n\tau\right)^{2}e^{-\bar{q}n\tau}, \end{matrix}$$

we find that the mean on-rate evolves as

$\tau_{0}\frac{d\bar{q}}{dt}=\sigma^{2}\frac{n\tau e^{-\bar{q}n\tau}}{\tilde{C}_{1}+1-e^{-\bar{q}n\tau}}$,

where $\tilde{C}_{1}=C_{1}(1+k/\lambda)$. The variance evolves as

$$\tau_{0}\frac{d\sigma^{2}}{dt}=2D+\frac{\mathrm{cov}\left(\left(q-\bar{q}\right)^{2},A\right)}{\left\langle A\right\rangle+C_{1}}\approx2D+\frac{A_{2,\bar{q}}}{\left\langle A\right\rangle+C_{1}}\sigma^{4}=2D-\sigma^{4}\frac{\left(n\tau\right)^{2}e^{-\bar{q}n\tau}}{\tilde{C}_{1}+1-e^{-\bar{q}n\tau}}.$$

Thus, similarly to the two arms case, the mean on-rate and variance evolve according to a coupled non-linear system. Again, the above equations are valid only when $\bar{q}$ is small. We plot the numerical solution of the equations and in S7 Fig. Interestingly, when $\tau$is of the same order on the effective on-rate (basal on-rate multiplied by the amount of Ag molecules), the mean affinity has a maximum at intermediate densities (S7bc Fig). However, $\tau$ is likely much larger than the on-rate, in which case the affinity is monotonously decreasing as a function of the Ag density (see S7de Fig).

## References

1. Schuss Z. Diffusion and Stochastic Processes. An Analytical Approach. New York, NY: Springer-Verlag; 2009.

2. Smerlak M, Youssef A. Limiting fitness distributions in evolutionary dynamics. J Theor Biol [Internet]. Elsevier; 2017;416[December 2016]:68–80. Available from: http://dx.doi.org/10.1016/j.jtbi.2017.01.005
